# A far from equilibrium state developed from pulse poling for high performance piezoelectric transducers

**DOI:** 10.1186/s40580-025-00528-8

**Published:** 2025-12-19

**Authors:** Michael W. Mervosh, Ju-Hyeon Lee, Clive A. Randall

**Affiliations:** 1 Department of Material Science and Engineering, The Pennsylvania State University, N-244, Millennium Science Complex, University Park, PA 16802 USA; 2 Center for Dielectrics and Piezoelectrics, Materials Research Institute, University Park, PA 16802 USA

## Abstract

Creating a piezoelectric or pyroelectric material from a ferroelectric material requires aligning its ferroelectric domains to achieve a remanent polarization. This complex process involves nucleating/growing domain structures and inducing a macroscopic, non-centrosymmetric symmetry under an applied electric field. For many years, this process has not received significant attention. Typically, DC fields are applied at elevated temperatures to align the domain states, balancing the depolarization and screening fields in a metastable state that is still near equilibrium. In contrast, the pulse poling (PP) strategy uses field pulses much faster than the time it takes for depolarization and bulk screening processes to occur. This instability allows the PP of relaxor ferroelectric (RFE) crystals and textured ceramics to induce a new far-from-equilibrium (FFE) state, which has enhanced properties compared to conventional DC poling. RFE crystals are of interest because it is known that poling them in specific directions creates engineered domain structures that provide giant piezoelectric properties with low hysteretic losses. By applying PP to RFE crystals in the < 001 > direction, significant changes in the electromechanical properties within the FFE state were observed, which opens new high-performance opportunities for these materials in transducer applications. This review outlines the property enhancements with PP and their origins while modeling the properties with a phenomenological thermodynamic approach. The properties are discussed with respect to transducer applications and benchmarked to other traditional poling strategies. Mn: PMN-PIN-PT, Mn: PMN-PZT, and Sm: PMN-PIN-PT are primarily used as model systems to demonstrate enhanced electromechanical performance with PP over other conventional poling strategies. Given the new concepts discussed in this paper, there is also a future research section at the end of the paper to drive the innovation beyond this initial work.

**Graphical abstract:**

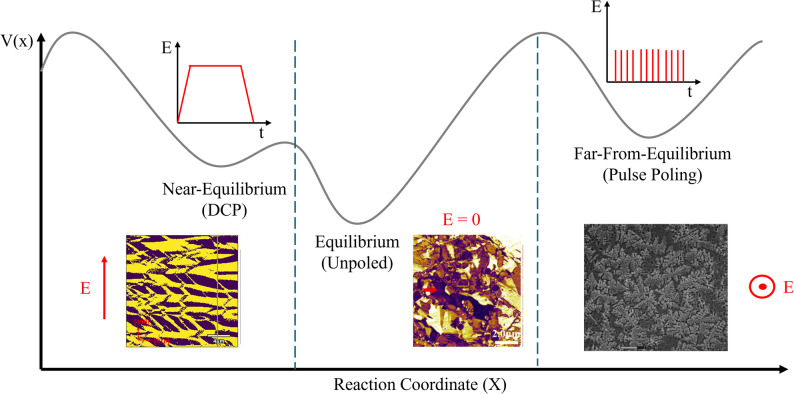

## Relaxor ferroelectric (RFE) materials

### Relaxor ferroelectric (RFE) materials and domain engineering

**Fig. 1:**
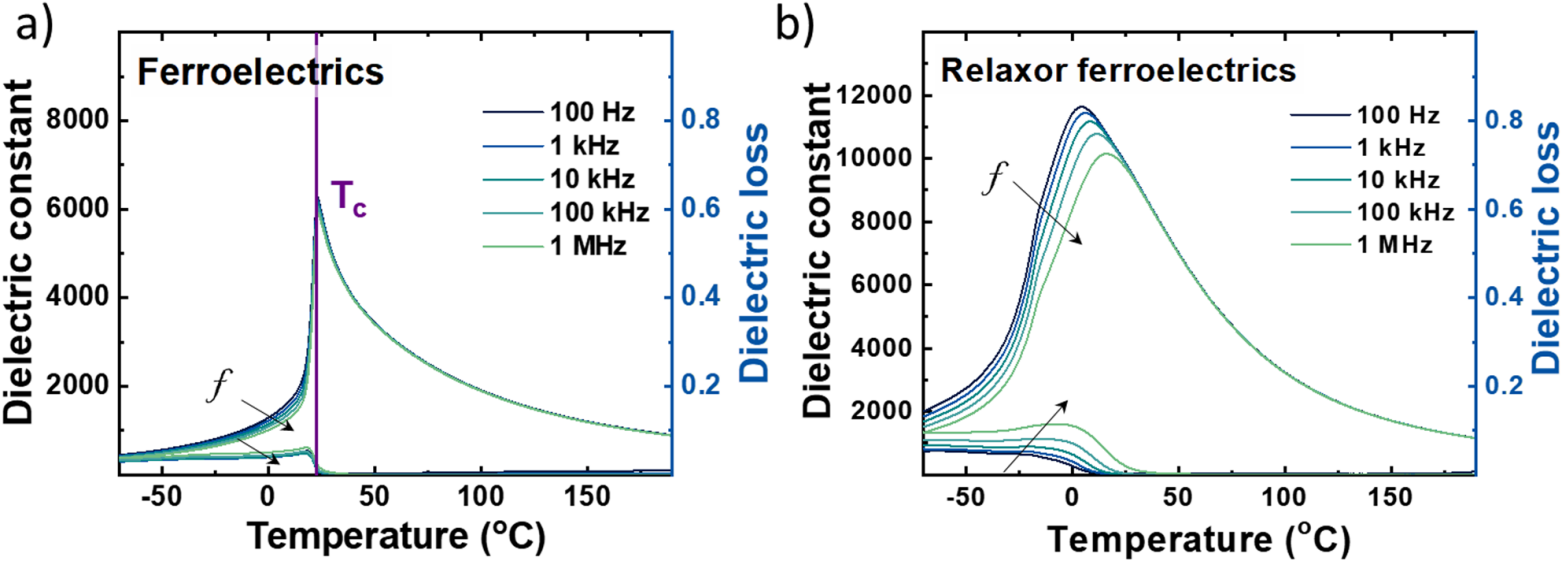
Temperature, frequency-dependent dielectric permittivity and loss of (**a**) conventional ferroelectrics and (**b**) relaxor ferroelectrics (Data in (**a**) were obtained from a B-site-ordered PbSc_1/2_Ta_1/2_O_3_, while (**b**) reflects the dielectric response of a B-site-disordered PbSc_1/2_Ta_1/2_O_3_ material)

A relaxor ferroelectric is a type of ferroelectric material with a unique set of properties, including a diffuse phase transition and a highly frequency-dependent dielectric constant [1–4]. Unlike conventional ferroelectrics that exhibit a sharp, single-temperature phase transition, relaxors have a broad transition range (Fig. 1). This behavior is linked to the presence of disordered, nanoscale polarized regions within the material, rather than the large, ordered domains found in normal ferroelectrics [5–8]. This disorder leads to the formation of polar nanoregions (PNRs) and results in an extremely large dielectric constant. One of the first and most notable examples is lead magnesium niobate (PMN), reported in the 1960s [4]. These materials have a broad diffuse phase transition with frequency-dependent permittivity associated with the dynamic and frustrated interactions of PNRs [9–11].

To enhance their properties, these RFE materials can form solid solutions with classical ferroelectric materials like lead titanate (PT) [3, 12]. This combination creates a morphotropic phase boundary (MPB) region, around which the materials exhibit ultrahigh dielectric and piezoelectric properties [13]. These compositions are based on materials with complex B-site chemistries in the octahedral site of the perovskite structure, with Pb cations occupying the 12-fold coordinated A-sites in the general ABO_3_ unit cell [2, 14]. B-site chemical ordering can occur in these relaxor compounds, which leads to lattice frustrations that perturb the polar coupling of the ferroelectric polarization ordering, this chemical ordering is diluted with PT content [10, 11]. Binary relaxor ferroelectric solid solutions are formed between Pb(B’B’’)O_3_ compounds with PbTiO_3_. Examples of this include (1-x)Pb(Mg_1/3_Nb_2/3_)O_3_ − xPbTiO_3_ (PMN-PT) and (1-x)Pb(Zn_1/3_Nb_2/3_)O_3_ − xPT (PZN-PT), where 0 ≤ x ≤ 1.

**Fig. 2:**
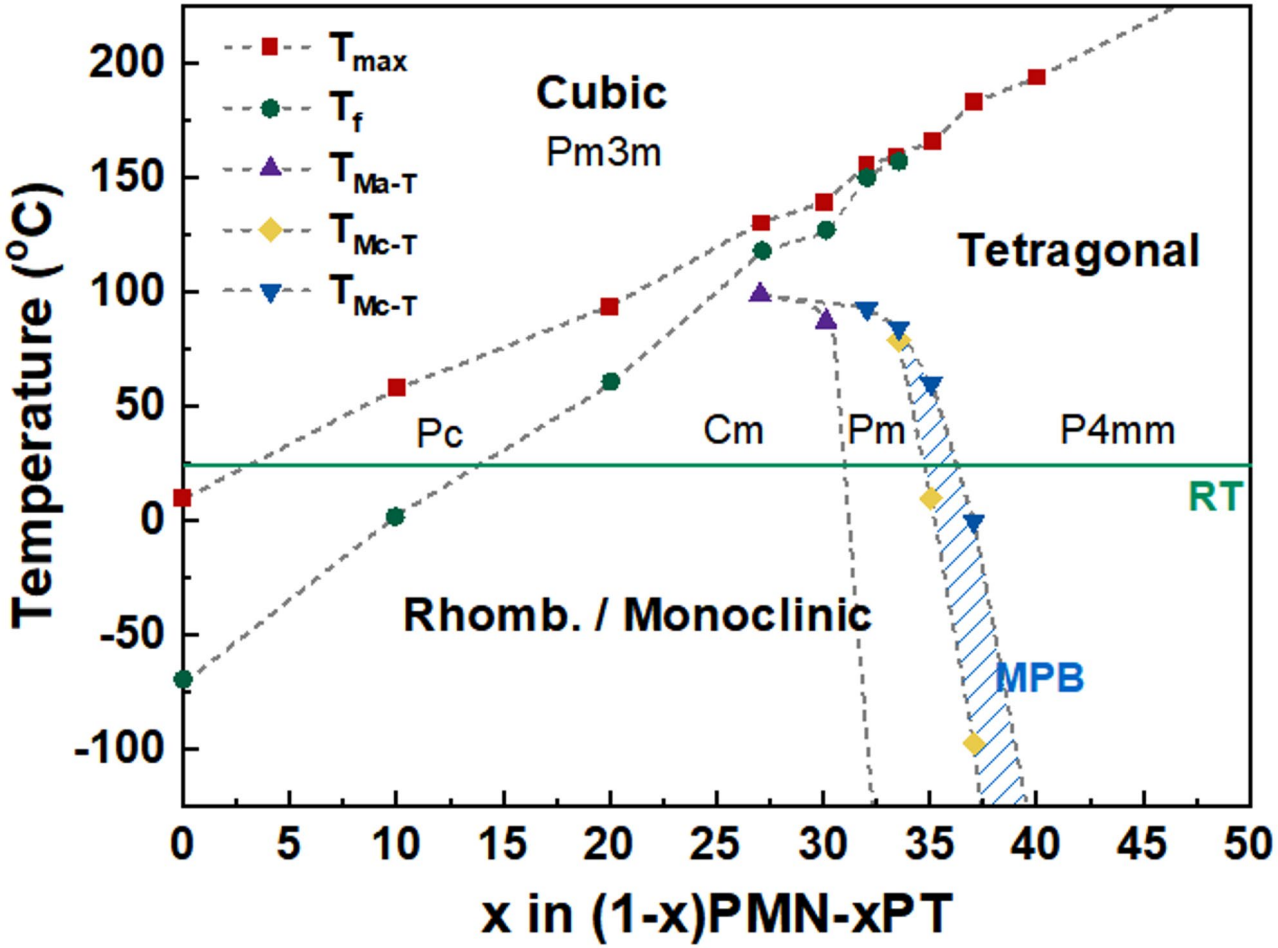
Phase diagram for a (1-x)PMN-xPT relaxor with monoclinic, rhombohedral, and tetragonal ferroelectric phases around MPB and a high temperature paraelectric phase. (adapted from [15])

A typical phase diagram of PMN-PT is shown in Fig. 2 [15]. The PT side of the phase diagram is a relatively simple with a tetragonal P4mm normal ferroelectric phase. On the relaxor side, there is a complex mixture of an averaged rhombohedral ferroelectric phase with different monoclinic distortions [15]. At high temperatures is a paraelectric phase with Pm3m symmetry. The application of an electric field in the relaxor ferroelectric phase can readily perturb the PNRs and drive the material to have much longer-range order. This was originally referred to as a macrodomain state, which reflects anisotropic properties not easily observed in the PNR state which is unpoled [4, 16]. The macrodomain state can be destabilized by heating the materials to higher temperatures, where depolarization results in a conversion back to the relaxor state. As compositions approach the MPB, the degree of relaxor and normal ferroelectric behavior changes, and the relaxor character will lessen, causing the system to tend towards a normal ferroelectric with more macroscopic ferroelectric domains. There is, however, residual relaxor character reflected in the dispersion of unpoled dielectric permittivity versus temperature in the radio frequency range. It is solid solution compositions in this intermediate compositional region that are of interest for piezoelectric applications, owing to the giant piezoelectric properties in single crystals and textured ceramics with engineered domain states described later on [17–19].

Classic polycrystalline piezoelectric ceramics, like Pb(Zr_1-x_,Ti_x_)O_3_, start in an unpoled, symmetrical state (∞∞m Curie symmetry) with an inversion center. When a sufficiently high electric field is applied, they can be poled, losing their inversion center and changing to a macroscopic ∞m symmetry [20]. This change allows them to exhibit piezoelectric activity as they become polar and non-centrosymmetric. In contrast, RFE single crystals can be poled along different crystallographic directions, which results in various macroscopic symmetries, such as 4 mm, mm2, and 3 m [21–24]. This leads to strong anisotropic characteristics, meaning their properties vary depending on the direction. This process creates engineered domain configurations for ferroelectric crystals where a crystal is poled along a non-zero-field polar axis. This reorients the crystal's polarization vectors to minimize their angle to the poling direction, creating a specific set of domains.

**Fig. 3:**
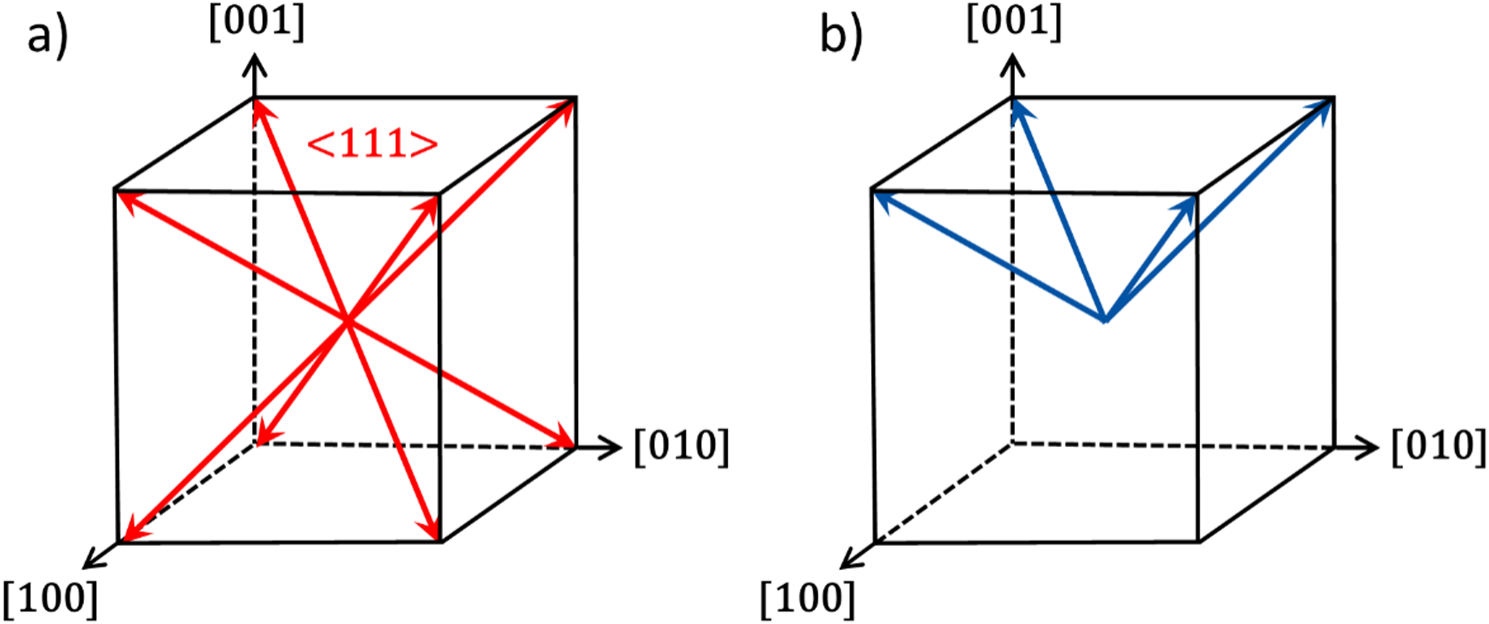
**a** All rhombohedral orientations < 111 > represented in an unpoled RFE crystal composition relative to the cubic axes, **b** a poled representation when the poling electric field was in the [001] direction

If one considers the average rhombohedral (R) ferroelectric phase in an RFE based composition, they can examine the different domain configurations aligned relative to each major crystallographic orientation: < 001 > , < 011 > , and < 111 > [23, 25, 26]. When poled along these different directions, these crystals form different macrodomain configurations, each with unique properties. A schematic of these configurations is shown in Fig. 3. [001]-poled crystals form a 4-domain configuration, [011]-poled crystals form a 2-domain configuration, and [111]-poled crystals form a 1-domain configuration (a single-domain state) [25]. In most recent pulse poling investigations, the [001] cases have been considered.

Within the engineered domain state, the field orientation relative to the domain polarizations allows for easy rotation of the spontaneous polarization vectors towards the applied electric field [22–24]. This rotation is a continuous process that doesn’t require the irreversible, energy-dissipating motion of domain walls. This mechanism, often referred to as polarization rotation, is considered a major reason for the giant piezoelectricity and high electromechanical coupling observed in these RFE crystal materials [27]. By minimizing the extrinsic, hysteretic effects of domain wall motion, the intrinsic, high-efficiency response is maximized. There are, however, still irreversible domain wall contributions in these systems, as have been modeled with phase-field methods [28, 29]. Experimental Rayleigh analysis on RFE-poled crystals also provides experimental evidence of an irreversible domain contribution, within the RFE crystals [30, 31]. The < 001 > textured materials have non-ideal alignments between adjacent textured grains, which can lead to lossy electromechanical processes and higher hysteretic losses.

### Compositional design of relaxor ferroelectrics materials for piezoelectric crystals

RFE crystals are of interest for transducers used in medical ultrasound diagnostics, non-destructive testing, energy harvesting, and sonar. This interest stems from the early work from Kuwata, Uchino, and Nomura showing high piezoelectric properties in the < 001 > direction and later a more comprehensive investigation with Park and Shrout [13, 32]. Since the demonstration of giant piezoelectric properties from these crystals, there has been continuous progress in crystal growth methods and compositional development to enhance the electromechanical properties for transducer applications. There are three main families of RFE crystals and textured ceramics: Gen I, Gen II, and Gen III [33].

**Gen I** materials are first-generation RFE single crystals based on binary solid solutions with compositions near the so-called morphotropic phase boundary (MPB) [2, 12]. This includes systems such as (1 − x)PMN − xPT and (1 − x)PZN − xPT. The MPB is a specific compositional region in the phase diagram of a solid solution where two or more different ferroelectric crystal phases coexist. There is a large enhancement of properties in these compositional regions. In RFE systems, a curved MPB can cause a phase transition between phases with increasing temperature, potentially limiting the operational temperature range [26, 34–36]. Enhanced temperature sensitivity of properties and depoling due to a curved MPB are problematic for transducers having to operate under different ambient conditions.

**Gen II** materials are second-generation systems that sought to raise phase transition temperatures while still enhancing properties. These are ternary systems like xPb(In_1/2_​Nb_1/2_​)O_3_​ − yPMN–zPT (PIN-PMN-PT), with x + y + z = 1. These ternary systems increase T_c_, the MPB transition, T_RT_, and the coercive field (E_c_).

**Gen III** materials are third-generation RFEs doped with acceptors such as Mn to create internal fields and harden the properties [33]. Gen III crystals have higher coercive fields to enhance stability under high-drive conditions and also have higher Mechanical Quality Factors (Q_m_).

## Single crystal, solid state crystal, and templated grain growth

### Bridgman crystal growth

**Fig. 4:**
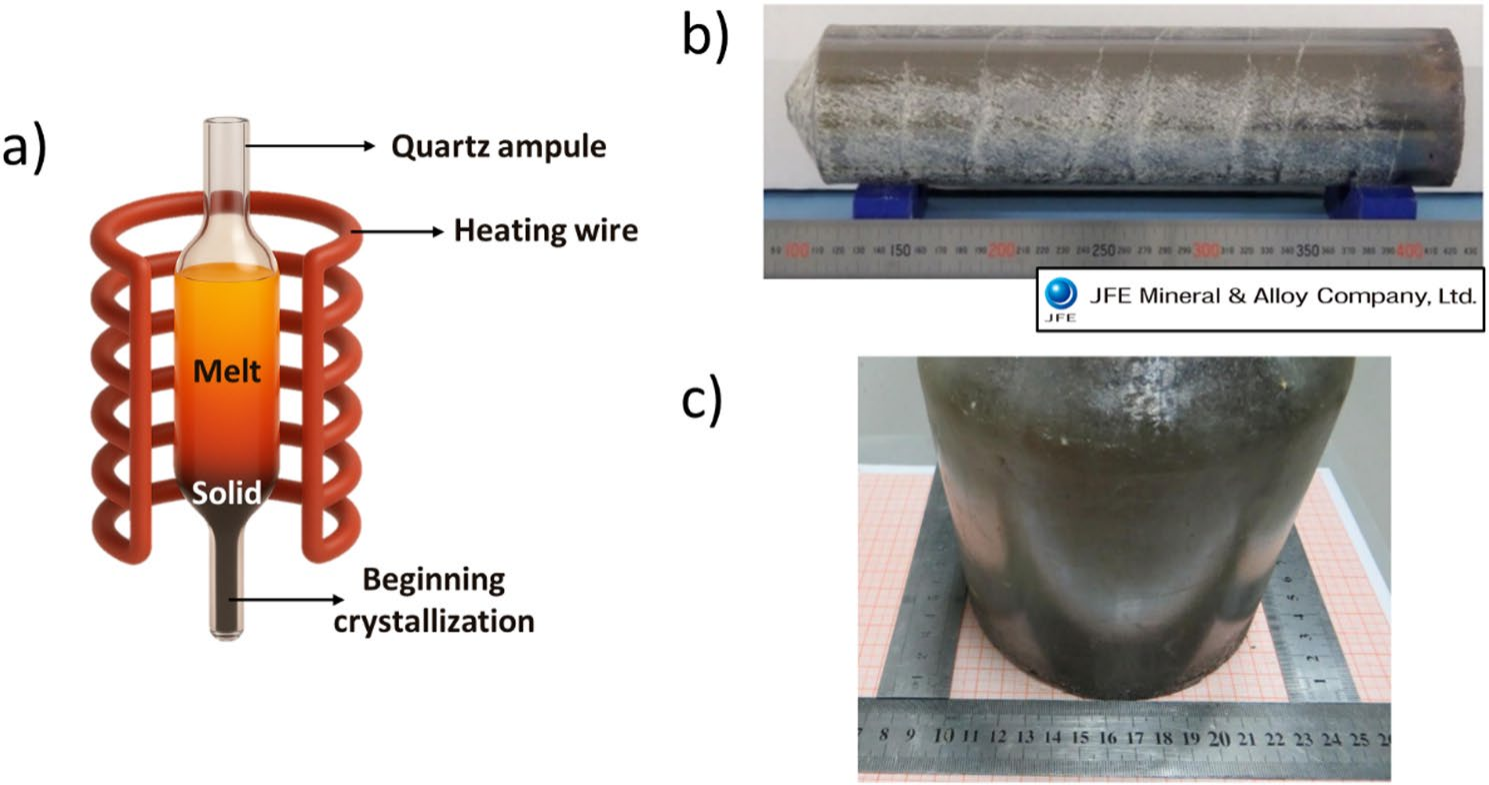
**a** Schematic of the Bridgman method, **b** Photo of PMN-PT single crystal grown by the Bridgman method from JFE Mineral & Alloy Company [38] Ltd, and **c** Photo of as grown crystal of PIN-PMN-PT (adapted from [17] under CC-BY-NC license)

The ability to engineer domain states in different directions in single crystals led researchers to commercialize effective growth methods for producing RFE crystals. Bridgman Crystal Growth has proven to be the most advanced commercial approach [17, 18, 25, 37]. The Bridgman-Stockbarger technique, named after its inventors Percy Bridgman and Donald Stockbarger, is a common method for growing single crystals. This technique uses a furnace with a controlled temperature gradient to promote directional solidification. The process begins with a polycrystalline ceramic precursor, which melts in the hot zone of the furnace. A seed crystal, cut to a specific orientation, is then brought into contact with the molten material at the bottom of the crucible. The crucible is then slowly moved from the hot zone into the cooler part of the furnace. This gradual translation allows for a continuous and controlled growth of a single crystal from the seed [18, 37]. In some designs, the crucible is fixed, and the furnace moves, while in others, the furnace is fixed, and the crucible moves. Both vertical and horizontal configurations exist. At the time of this writing, the leading manufacturers of RFE crystals (JFE Mineral and Alloy Company, Ltd., TRS Technologies, and CTS) are all pushing larger diameter crystals between 7.5 to 10 cm with the Bridgman method (Fig. 4) [17, 38].

### Solid state crystal growth (SSCG)

**Fig. 5:**
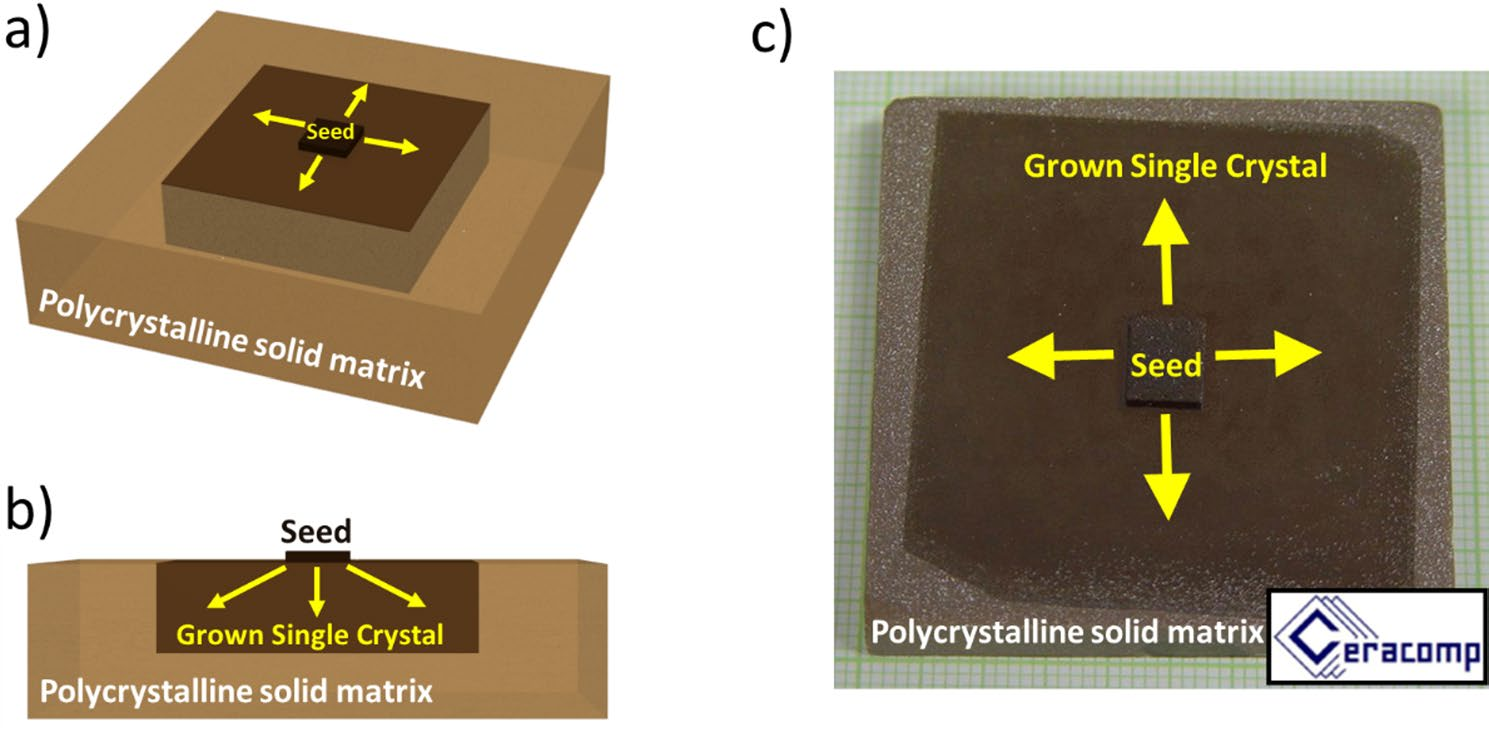
**a**-**b** Schematic of SSCG method, **c** Photo of PMN-PT single crystal grown by SSCG method from CeraComp [41]

Solid State Crystal Growth (SSCG) is a method that intentionally uses a phenomenon called abnormal grain growth to produce single crystals from polycrystalline ceramics. Normally, abnormal grain growth is considered a problem in ceramics because it can lead to uncontrolled material properties and reduced reliability [39]. However, SSCG harnesses this process. First, a dense, fine-grained polycrystalline material is prepared. Then, a small single-crystal seed is attached to this ceramic. The combined material is heated to a temperature well above the normal sintering temperature but still below the melting point of the material. At this high temperature, the large single-crystal seed begins to "grow" into the surrounding polycrystalline material, effectively converting the fine-grained ceramic matrix into a single crystal [40]. Several factors can be adjusted to control the SSCG process, including temperature, surface finish, interface layers, thermal gradient, and atmosphere. Despite the interesting properties and chemical homogeneity that can provide advantages over Bridgman crystal growth, there are pores in the crystals from the incomplete densification of the matrix ceramic phase. There is also currently only one company that is supplying SSCG RFE crystals, CeraComp (Fig. 5).

### Templated grain growth (TGG)

**Fig. 6:**
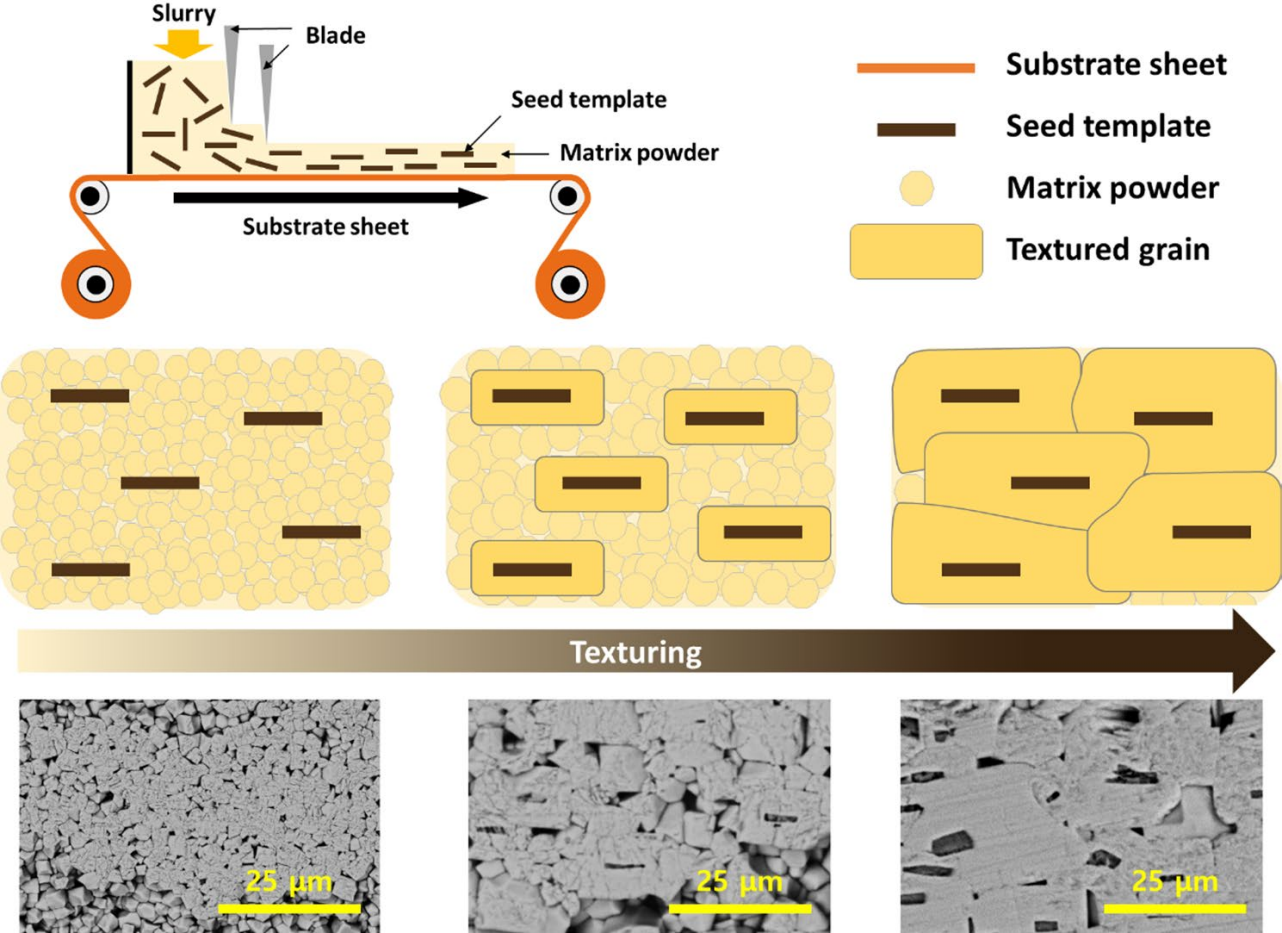
Schematic demonstration of the TGG process, along with the template in a polycrystalline matrix before and after the texturing transformation

TGG has the potential to enhance the performance of ceramic materials by creating anisotropic microstructures that can approach single-crystal performance in a chosen direction [19]. In the case of ferroelectrics, the texture direction is often selected to maximize the piezoelectric response through polarization elongation or polarization rotation [22, 24, 42]. In other cases, the texture direction is chosen to enable or disable mechanical deformation modes [43]. The schematic in Fig. 6 illustrates the typical process for creating textured piezoelectric ceramics. Tape casting technology, borrowed from the multilayer ceramic capacitor industry, is employed to produce a green ceramic tape containing highly oriented template particles. These larger, high-aspect-ratio particles serve as nuclei for the growth of the oriented microstructure by transporting material from the surrounding matrix to the crystalline surfaces of the template particles [33, 39].

**Fig. 7:**
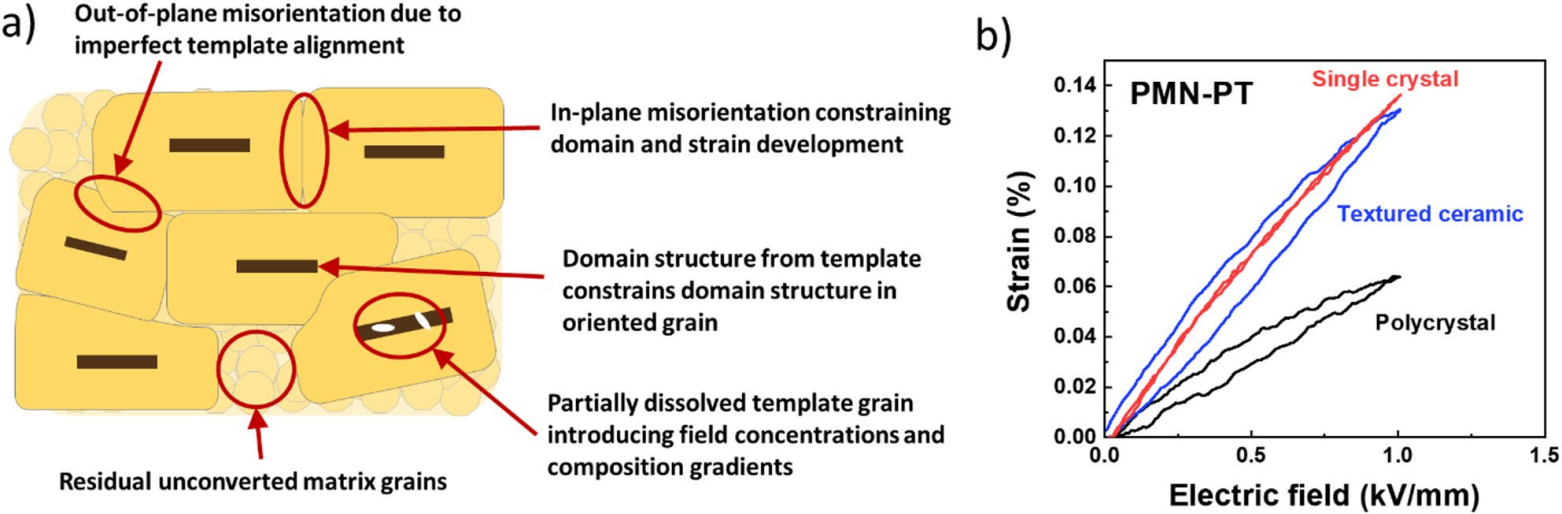
**a** Misalignment and incomplete texturing that can limit the textured materials, **b** high field hysteretic loss common to textured ceramics (replotted graph based on data from [46])

Crystallographic texture in piezoelectric ceramics enhances displacement and coupling relative to random ceramics. With texture fractions over 90%, the electromechanical response approaches 70–90% of single-crystal values [19]. These materials can advance performance in acoustic devices and greatly expand design space by offering size, shape, or performance characteristics not accessible by traditional random ceramics or single crystals. However, current materials, such as textured Mn: PMN-PZT and PMN-PT, have high dielectric losses and non-linear current–voltage behavior above 1–2 kV/cm [44, 45]. In contrast, in domain-engineered single crystals, these losses are substantially lower. Figure 7 demonstrates the non-ideal alignments that can arise between adjacent textured grains as well as the resultant hysteretic losses compared to single crystals [46]. The exact origin of these losses needs to be discovered and rectified before textured piezoelectric ceramics can be deployed for applications that require high fields and high duty cycles. However, it has been noted that under PP, the losses are suppressed relative to the DC-poled materials.

The ongoing goal of textured ceramics research is to tailor the processing and yield a material with properties beyond random polycrystalline ceramics and approaching that of single crystals. However, it is the question of scalability and production that will ultimately determine the value and commercialization of textured ceramics.

## Poling piezoelectric RFE crystals and textured materials

In the absence of an external electric field or strain gradient, ferroelectric materials have a macroscopic polarization of zero. This occurs because the spontaneous polarization within the domains and their local configurations self-cancel to minimize the overall elastic and electrostatic energy [47–49]. Upon the application of an external electric field above and near the coercive field (E_c_), a material’s domains will begin to align or nucleate new domains whose orientations are based on the symmetry relations of the current ferroelectric phase. Conventional direct current poling (DCP) applies a unipolar electric field to a material, often at elevated temperatures, to aid in domain mobility [50–52]. After poling, ferroelectric materials can exhibit pyroelectric and piezoelectric properties, with the latter involving a coupling of electrical and mechanical energy [50]. In recent years, several efforts have been made to improve the capabilities of DCP.

One of the most studied unconventional poling procedures for piezoelectrics is Alternating Current Poling (ACP) [53, 54]. ACP switches the direction of the poling field at a given frequency for a specific number of cycles. Prior to ACP’s popularization by Yamashita et al., previous studies, such as one by Ogawa et al., had shown the merits of applying bipolar fields to piezoelectric materials [54, 55]. They achieved large electromechanical property values in their PMNT system after applying bipolar fields for the purpose of collecting hysteresis loops [56]. It has been hypothesized that this method reduces the pinning effect during polarization reversal. In addition, some researchers argue that the fine, striped domains in appropriately oriented crystals are responsible for the 20–50% increases in piezoelectric properties relative to DCP [53, 57].

However, a vast majority of ACP work has been conducted on single-crystal samples as opposed to textured ceramics. The few studies on textured ceramic ACP report modest gains, including one paper by Yang et al. claiming only a small 6% increase in piezoelectric coefficient ​ and permittivity relative to DCP [58].

**Fig. 8:**
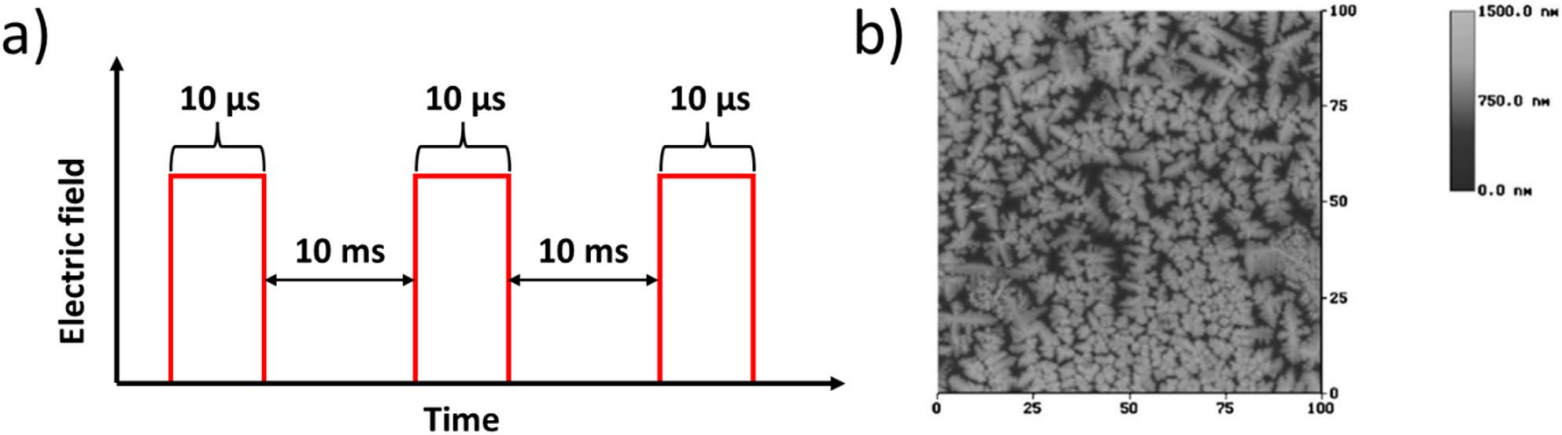
**a** Schematic of Pulse Poling (PP), and **b** Atomic Force Microscopy image of dendritic domain structures after pulse poling and etching of (0.955)Pb(Zn_1/3_Nb_2/3_)O_3_-0.045PbTiO_3_. (obtained under CC-BY-NC license)

A third kind of poling, known as Pulse Poling (PP), subjects samples to short, rapid electric field pulses [59]. A schematic of this procedure is provided in Fig. 8 [45]. PP has mainly been used to periodically pole lithium niobate (LiNbO_3_) for optical applications [60]. An earlier study at Penn State (Yu et al.) showed the ability to improve the piezoelectric coefficient of rhombohedral (1-x)PZN − xPT single-crystal samples by about 30% using 10 μs pulses [59]. More recent PP efforts have shown even greater property improvements relative to DCP, and ACP (as high as 65–75% greater) through a two-stage pulsing strategy [45].

**Fig. 9:**
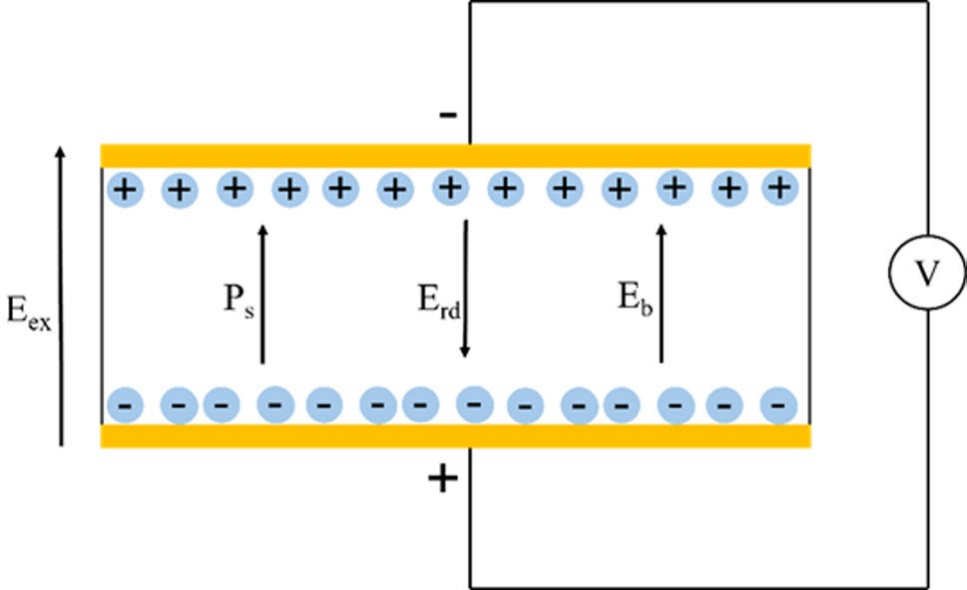
Field dynamics during the poling process with compensating fields E_rd_ (the depolarization field) and E_b_ (the bulk screening field)

The Yu et al. study and previous lithium niobate studies reported the formation of dendritic domains in their samples from pulse poling [60–62]. An example of such domains is given in Fig. 8b [59]**.** This contrasts with other PP efforts using wider pulse widths (0.1–0.2 s), which did not report these dendritic domains [63, 64]. An explanation for this is offered by Shur et al. in their LiNbO_3_ work: they claim that dendritic domains are likely to form if there is an ineffective screening of the depolarization field [60]. Such competition between the various fields arises from the complex field dynamics that develop during poling, as shown in Fig. 9 [65].

Whether a favorable domain will nucleate during poling is governed by the magnitude of the local electric field (E_loc_) in each region [60, 66]. This value depends on the competing effects between the applied external field (E_ex_), bulk screening field (E_b_), and residual depolarization field (E_rd_). The resultant expression for E_loc_ is given by [60]:

3.1 $$ {\text{E}}_{{{\text{loc}}}} = {\text{E}}_{{{\text{ex}}}} + {\text{ E}}_{{{\text{rd}}}} + {\text{ E}}_{{\text{b}}} $$

For the purpose of dendritic domain growth, the most relevant contribution is the degree to which the depolarization field, E_rd_, is screened by E_b_. This is best described by R, the ratio between the field switching rate (1/t_s_) and the bulk screening rate (1/τ_sc_) [60]:

3.2 $$ {\text{R}} = \tau_{{{\text{sc}}}} /{\text{t}}_{{\text{s}}} $$

**Fig. 10:**
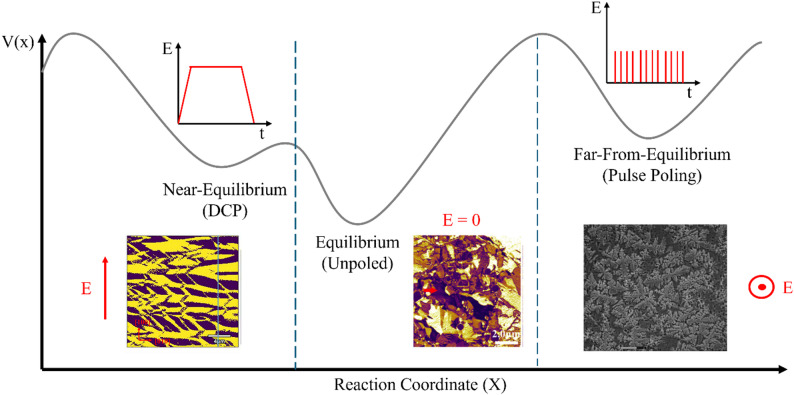
Schematic representation of the potential energy landscape of poling procedures with corresponding domain structures and poling direction. (Figure modified with data from [61, 68])

An R <  < 1 yields quasi-equilibrium conditions with effective screening, an R ~ 1 yields off-equilibrium conditions with incomplete screening, and an R >  > 1 yields non-equilibrium conditions and ineffective screening [60]. The bulk screening process involves multiple contributions, including moving bulk charges, reorienting defect dipoles, and injecting charge from the surface electrodes [62]. The time scales for these mechanisms range from milliseconds to months. Thus, the microsecond or less switching times employed by some pulse poling studies would generate a large R value and ineffective screening. The studies using wider pulse widths would diminish their R constants and likely fall outside of this R threshold, causing their materials not to form the dendritic domain structures characteristic of non-equilibrium poling conditions. Under such conditions, in conjunction with elevated pulsing temperatures, the system is being pushed into a far-from-equilibrium (FFE) state, which allows for different behavior than would otherwise be expected in a near-equilibrium state, as schematically shown in Fig. 10 [67].

When pulse poling, it has been found that a 2-stage pulse poling procedure is the most effective [45]. The first stage applies low field domain nucleation-controlled pulses, and the second applies high field growth-controlled pulses. The fields in which this promotion efforts take effect are based on prior work investigating dendritic domain formation in PZN-PT single crystals [59, 61]. Often, it helps to operate at elevated temperatures near the T_RT_ transition in what is known as high temperature pulse poling (HTPP). After applying the high temperature pulses, the materials are cooled under a DC bias to prevent domain back switching. Table 1 details the materials showcased in this paper as well as the poling procedures employed for the following property analyses.

**Table 1:** Material information and poling conditions

Material:	Microstructure:	Material Type:	Poling Procedure:	Poling Conditions:
Mn: PIN-PMN-PT	Single Crystal (Bridgman)	Gen III (Hard)	DCP	Field of 7.5 kV/cm at 80 °C for 15 min
Mn: PIN-PMN-PT	Single Crystal (Bridgman)	Gen III (Hard)	ACP	Field of 10 kV/cm, at 0.1 Hz for 10 cycles using triangular waveform
Mn: PIN-PMN-PT	Single Crystal (Bridgman)	Gen III (Hard)	Pulse Poling	100 pulses 10 μs wide of 10 kV/cm followed by 100 pulses of 20 kV/cm at 80 °C
Sm: PIN-PMN-PT	Single Crystal (Bridgman)	Gen II (Soft)	DCP	Field of 7.5 kV/cm at 25 °C for 15 min
Sm: PIN-PMN-PT	Single Crystal (Bridgman)	Gen II (Soft)	ACP	Field of 10 kV/cm, at 1 Hz for 10 cycles using triangular waveform
Sm: PIN-PMN-PT	Single Crystal (Bridgman)	Gen II (Soft)	Pulse Poling	100 pulses 10 μs wide of 8 kV/cm followed by 100 pulses of 18 kV/cm at 80 °C
Mn: PMN-PZT	Textured Ceramic	Gen III (Hard)	DCP	Field of 25 kV/cm at 120 °C for 15 min
Mn: PMN-PZT	Textured Ceramic	Gen III (Hard)	ACP-FC	Field of 15 kV/cm, at 0.04 Hz for 50 cycles using triangular waveform
Mn: PMN-PZT	Textured Ceramic	Gen III (Hard)	Pulse Poling	100 pulses 10 μs wide of 6 kV/cm followed by 100 pulses of 25 kV/cm at 120 °C

## RFE electromechanical properties of importance to transducer applications

For a high-performance piezoelectric material to be considered state-of-the-art for transducers, several electromechanical figures of merit are crucial [69–72]. These parameters dictate how efficiently and effectively the material converts electrical energy to mechanical energy (actuation) and vice versa (sensing), as well as its stability and ability to handle high power. Each of these key properties is summarized below.

*Piezoelectric Constant (d)*: This constant quantifies the mechanical strain produced per unit of applied electric field, or conversely, the electric charge generated per unit of applied mechanical stress [73]. The piezoelectric coefficient is a third-rank tensor, often expressed in matrix notation with two subscripts specific to different stress or strain directions. For actuators, a high d value (e.g., d_33​_ for strain in the polarization direction with an electric field in the same direction) is desired for larger mechanical displacements or forces. When operating as a sensor, a large d coefficient provides a higher charge output for a given mechanical input, leading to greater sensitivity.

*Piezoelectric Voltage Constant (g)*: This constant describes the electric field generated per unit of applied mechanical stress, or the mechanical strain produced per unit of applied electric displacement. For sensors, a high g value (e.g., g_33_) is desirable as it indicates a larger voltage output for a given mechanical input, with stress applied to the polarization. The relationship g_33_ = d_33_/ε_o_ε_33_ highlights the interplay between d_33_ and the relative dielectric permittivity of the poled material, ε_33_, parallel to the polarization. Traditionally, it is difficult to decouple the piezoelectric coefficient from the high permittivity of a ferroelectric material, and therefore, composite structures with low-permittivity polymers are used to enhance the piezoelectric voltage coefficient [73].

*Electromechanical Coupling Coefficient (k)*: This is a crucial parameter, representing the efficiency of energy conversion between electrical and mechanical forms. A higher k value means a larger percentage of input electrical energy is converted into mechanical energy (and vice versa), leading to higher transducer efficiency, a wider bandwidth, and better sensitivity [73]. There are different coupling coefficients corresponding to various modes of vibration (e.g., k_33​_ for longitudinal vibration, k_p_ for planar coupling, k_t_ for thickness mode) [73]. A high electromechanical coupling coefficient is essential for high-performance transducers, particularly those for high power. The value of k can be conceptualized in the following way:

4.1 $$ k = {\text{ Stored}}\,{\text{Mechanical}}\,{\text{Energy }}/{\text{ Input}}\,{\text{Electrical}}\,{\text{Energy}} $$

The coupling coefficients are typically measured on the resonant structures, and *k* usually has to consider specific modes that are excited, the subscripts indicate these modes. Examples of these are given in the following equations [73]:

4.2 $${k}_{15}=\frac{{d}_{15}}{\sqrt{{s}_{55}^{E}{\varepsilon }_{11}^{X}}}$$

4.3 $${k}_{33}=\frac{{d}_{33}}{\sqrt{{s}_{33}^{E}{\varepsilon }_{33}^{X}}}$$

4.4 $${k}_{31}=\frac{{d}_{31}}{\sqrt{{s}_{11}^{E}{\varepsilon }_{33}^{X}}}$$

where d is the piezoelectric coefficient with matrix notation, ${\varepsilon }_{ij}^{X}$ is the dielectric permittivity (X denotes constant stress conditions), and ${s}_{ij}^{E}$ is the elastic compliance (E denotes constant electric field).

*Mechanical Quality Factor (Q*_*m*_*)*: This factor characterizes the sharpness of the electromechanical resonance and is inversely related to mechanical losses (damping) [73]. A high Q_m_ indicates low mechanical damping, which is critical for high-power transducers. Low losses reduce internal heating and allow the transducer to operate more efficiently at resonance, enabling a higher acoustic power output. The losses are typically associated with domain wall movement, and hence, the extrinsic contributions are important to control [73]. Even in engineered domain structures in crystals, there are finite contributions under DCP and with both Gen II and Gen III compositional designs. For continuous wave (CW) applications in the resonance mode, a high Q_m_ is often preferred, while for pulsed transmission applications, a lower Q_m_ might be acceptable for a broader bandwidth [73]. This is because Q_m_ is inversely related to the bandwidth of a transducer.

*Dielectric Loss Tangent (tan δ)*: The dielectric loss within a piezoelectric material indicates how much electrical energy is dissipated as heat when an electric field is applied [73]. A low dielectric loss tangent is crucial for high-power transducers to minimize heat generation, thermal runaway, and ensure efficient operation. High dielectric losses can lead to overheating and potential device failure through the depolarization of the component. The relation between dielectric loss and the real and imaginary parts of dielectric permittivity can be seen in:

4.5 $$ \tan \delta = \frac{{\varepsilon^{\prime \prime } }}{{\varepsilon^{\prime } }} $$

**Fig. 11:**
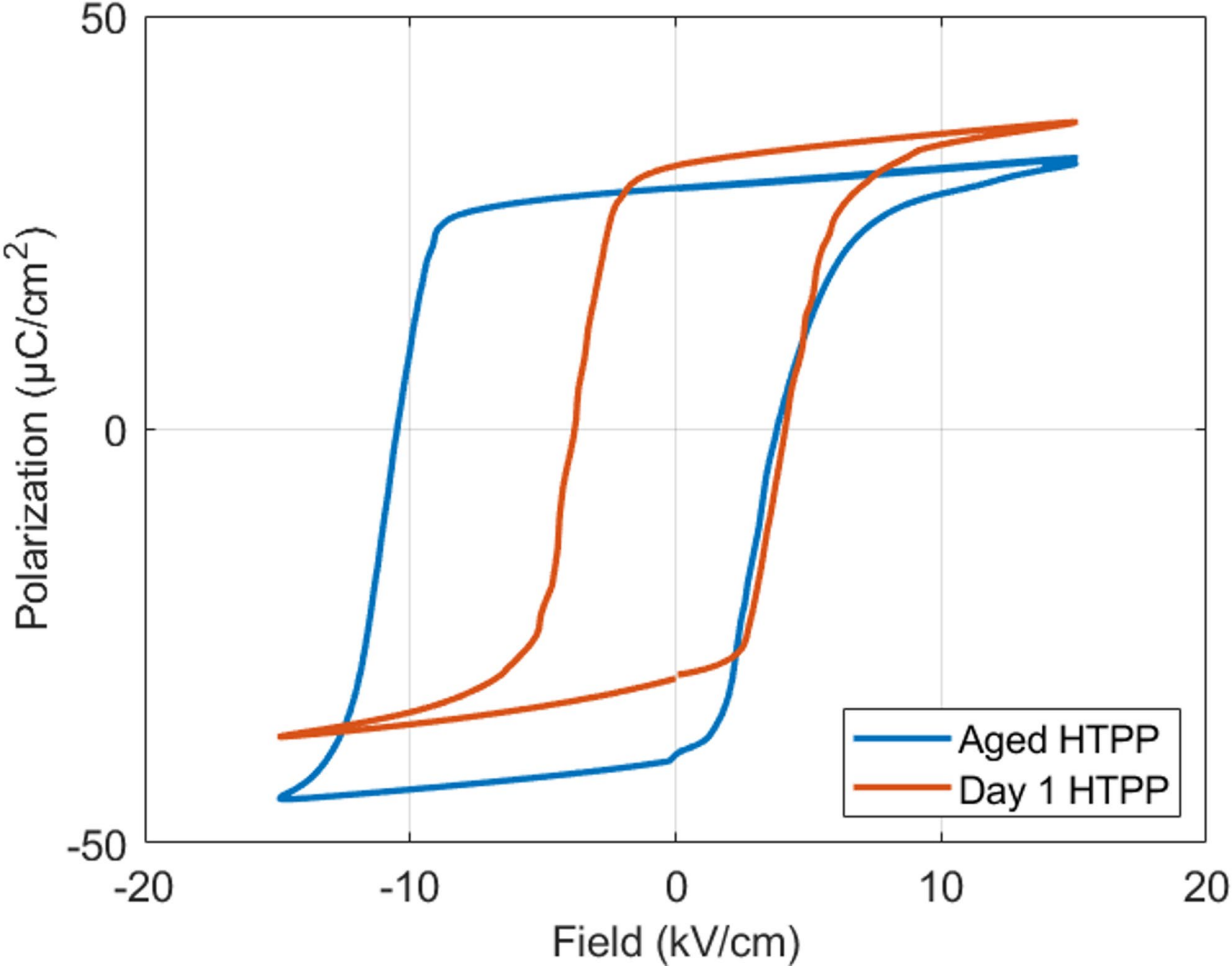
Hysteresis loop following the HTPP process in a Mn: PIN-PMN-PT crystal in [001] direction from 1 day to 1 month of aging

*Coercive Field (E*_*c*_*)*: This is the electric field required to depolarize a piezoelectric material after it has been poled [73]. A high coercive field indicates better domain stability and resistance to depolarization, which is important for high-power transducer operation and long-term reliability, especially under strong electric fields. Both the E_c_ and internal fields are important parameters in high-drive transducer materials. Figure 11 shows the remarkable change in the hysteresis of a Gen III RFE < 001 > crystal after PP and a month of positive aging; a large internal field develops, and there is also an increase in the coercive fields [65]. Some comparisons of the hysteresis properties with different materials and poling conditions are listed in Table 2 [65]. The internal field values within this table were calculated according to [74]:

**Table 2:** Measured internal field values of Mn-doped materials

Material	Poling Method:	E_c_(1), (kV/cm):	E_c_(2), (kV/cm):	E_int_ (kV/cm):
Crystal	Unpoled	7.1	− 6.15	0.20
Crystal	DCP	4.15	− 3.35	0.40
Crystal	HTPP (day 1)	4.10	− 3.70	0.20
Crystal	HTPP (aged)	3.65	− 10.6	3.48
Textured	Unpoled	6.74	− 6.09	0.32
Textured	DCP	5.76	− 8.25	1.24
Textured	HTPP (day 1)	7.43	− 6.90	0.27
Textured	HTPP (aged)	3.97	− 9.47	2.75

4.6 $${E}_{\text{int}}=\frac{{|E}_{c}\left(1\right)+ {E}_{c}(2)|}{2}$$

The internal field, E_int_, is determined from the difference between the forward and reverse coercive fields. For Gen III crystals and textured ceramics, the E_int_​ is based on compositions after HTPP and DCP. The E_int_ is twice as large in the textured ceramics after positive aging and HTPP, and almost an order of magnitude larger in the crystals, compared to the DCP case.

A comparison of the important electromechanical properties within the different materials and poling strategies is given in Table 3 [45]. These results are for samples subjected to ultra-fast pulses 10 μs wide. As was noted previously in this paper, there exist other, lower frequency pulse poling efforts employing 0.1–0.2 s pulses in the PIN-PMN-PT material family. These slower pulse poling efforts tend to outpace ACP with increases in d_33_ of about 50% [63, 64]. However, it is believed that there is a continuum of possible performance enhancement linked to the kinetics of the poling process in question. Through the use of ultra-fast pulse poling, a truly FFE state can be achieved with its associated large increases in single crystal performance relative to DCP, ACP, or slow pulse poling.

**Table 3:** Baseline electromechanical properties

Material:	Structure:	Poling Procedure:	d_33_ (pC/N):	Q_m:_	k_eff_:	ε_r_:	g_33_ × 10^–3^ (Vm/N):
Sm: PIN-PMN-PT	Single crystal	DCP	1,320	170	0.44	5,940	25.1
Sm: PIN-PMN-PT	Single crystal	Pulse Poling	2,200	120	0.76	9,600	25.9
Mn: PIN-PMN-PT	Single crystal	DCP	1,400	700	0.44	3,800	41.6
Mn: PIN-PMN-PT	Single crystal	Pulse Poling	2,500	500	0.79	3,700	76.3
Mn: PMN-PZT	Textured ceramic	DCP	940	770	0.49	2,070	51.3
Mn: PMN-PZT	Textured ceramic	Pulse Poling	850	950	0.5	2,190	43.8

**Fig. 12:**
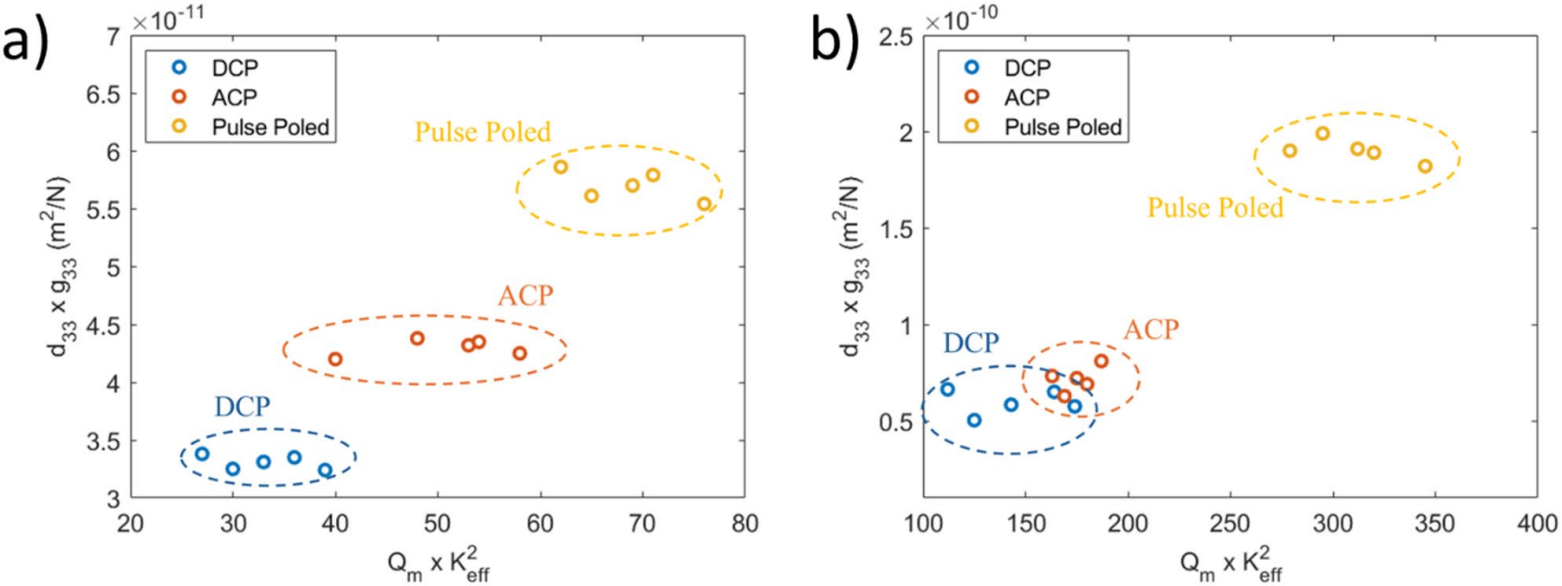
Comparison of the figures of merit for crystals [001] poled with DCP, ACP, and PP processes for **a** Mn: PIN-PMN-PT and **b** Sm: PIN-PMN-PT crystals

Overall, a high-performance piezoelectric transducer typically requires a ferroelectric material with a high electromechanical coupling coefficient, high piezoelectric coefficients, high mechanical quality factor, low dielectric loss, high coercive field, and high Curie temperature. These properties work together to deliver efficient, powerful, and stable transducer operation. Figure 12 shows a comparison of the figures of merit for the Gen II and Gen III versions of the same crystals poled via the DCP, ACP, and PP processes. Here the d_33_*g_33_ coefficient is plotted to reflect power generation in energy harvesting transducers, which is contrasted against k^2^*Q_m_ that impacts the acoustic power of the transducer.

**Fig. 13:**
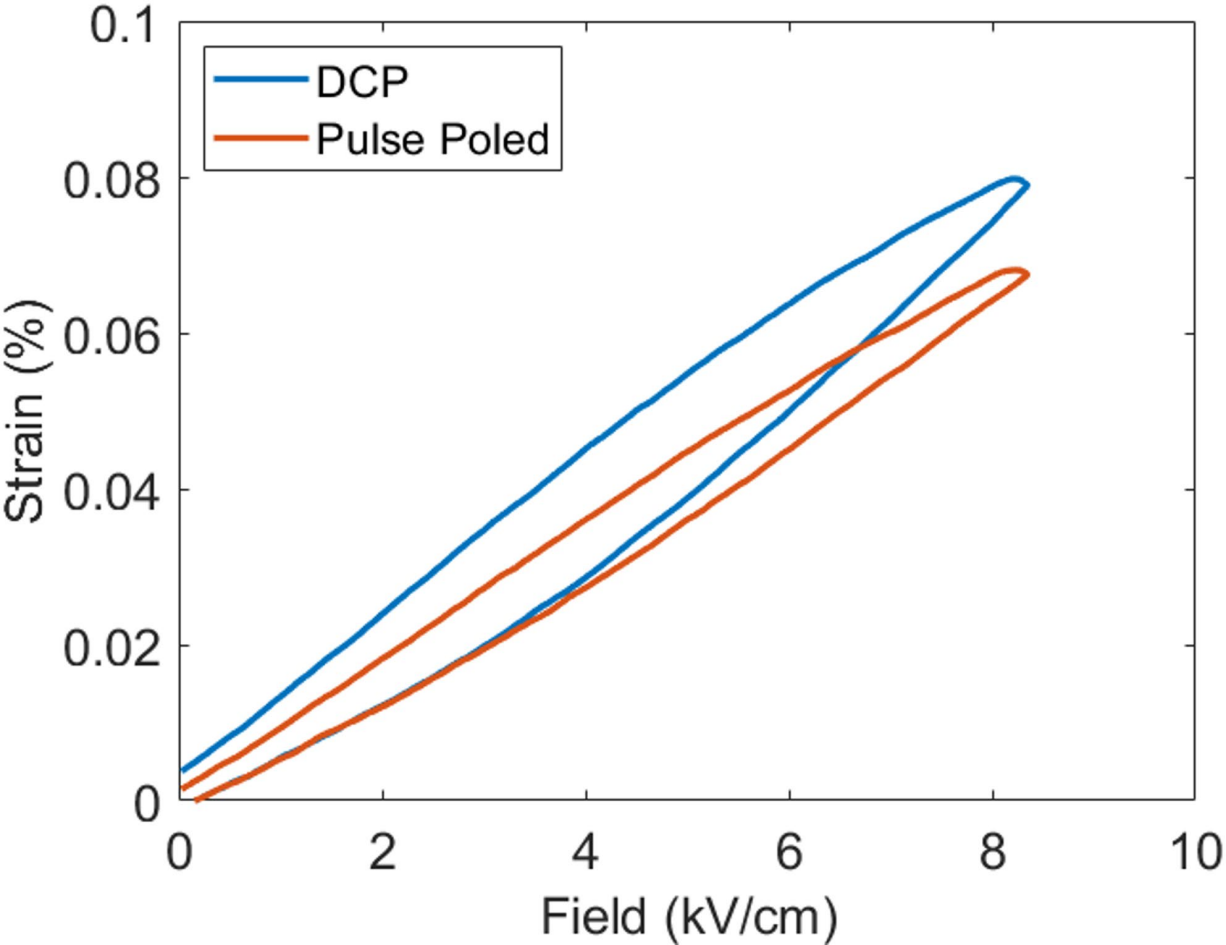
Strain-field measurements of Mn: PMN-PZT textured

In addition to the improved properties in the RFE single crystals, there are reduced hysteretic losses in the textured ceramics under the HTPP process [45]. In Table 3, there is a small loss in the magnitude of the piezoelectric coefficient in textured ceramics, but this is met with enhanced Q_m_ and electromechanical coupling under HTPP. This is also a very attractive observation and opportunity for textured ceramics. Figure 13 shows a strain field response on a textured ceramic material [45]. The lower hysteretic losses from reduced extrinsic contributions through HTPP relative to DCP are beneficial to textured ceramics meant for high drive transducer applications.

## Domain dynamics, switching, and property contributions

There are different aspects of ferroelectric domain behavior that are important for poling and a domain’s impact on properties. Ferroelectric switching and macroscopic polarization reversal (which forms a hysteresis loop) result from domains undergoing switch processes [2, 75]. Such processes include the nucleation of reverse polar domains and forward/sideways 2D growth of domain. Domains will reflect the symmetric relations of the high-temperature paraelectric phase, and in the perovskite-structured RFEs considered here, this is a cubic crystal symmetry with point group Pm3m [20]. The ferroelectric phases within the phase diagrams of the binary and ternary RFE compositions of interest are tetragonal (4 mm), rhombohedral (3 m), and monoclinic (m) [20]. The room-temperature phases are typically considered to be rhombohedral (R) for lower PbTiO_3​_ content and tetragonal (T) for high PbTiO_3_ [15]. As the temperature increases and crosses the phase boundary, there can be a phase transition from R to T, and in and around this region, an intermediate monoclinic phase (M) has been reported.

**Fig. 14:**
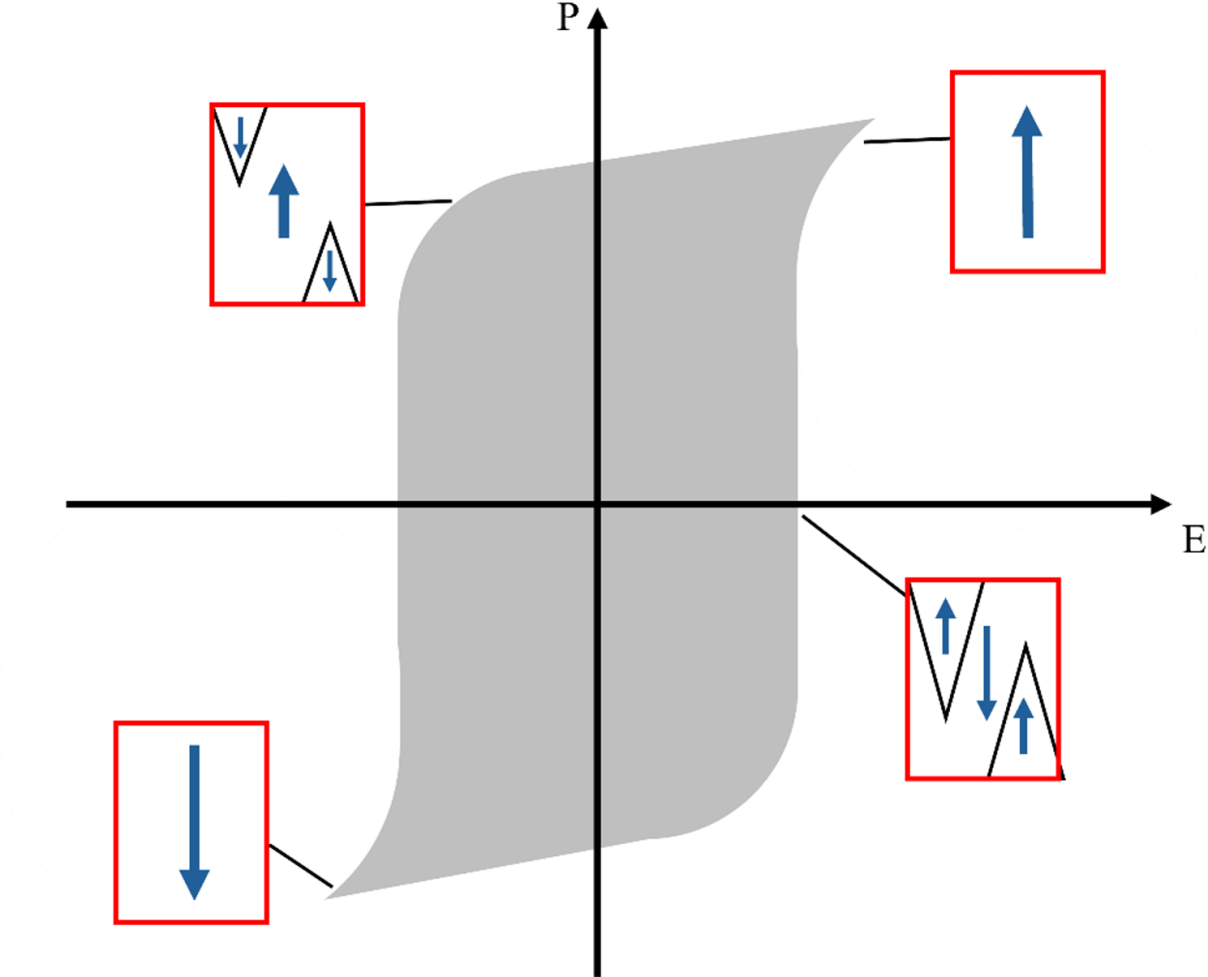
Nucleation and growth of domains during hysteresis loop switching of polarization under and AC electric field with amplitude exceeding the coercive fields

To access domain switching kinetics, revisiting the original work of Merz (1954) offers a useful first-order consideration of nucleation and domain wall movement for experimentally determining the field magnitudes and switching times to be used in PP [76]. Merz recognized that in a ferroelectric crystal, polarization reversal occurs through multiple nucleation and domain growth processes over a collective period of time [76]. When a DC field is applied to a crystal in a direction opposite to the spontaneous polarization, the polarization reversal begins with the formation of domain nuclei on random surfaces of the crystal in a statistical process. Nuclei that are over a critical size will continue to grow in the direction of the field until they become domains and reach the other side of the crystal [76]. Additional nuclei can form on existing domain walls and interfaces. There will also be sidewall movement to drive more regions to align with the applied field. To provide insights into the kinetic process, the switching processes will be illustrated in a much more simplified case for a uniaxial ferroelectric with just an inversion (180^∘^ domain system). Figure 14 shows a systematic view of switching through a hysteresis loop with nucleation and domain wall movement.

**Fig. 15:**
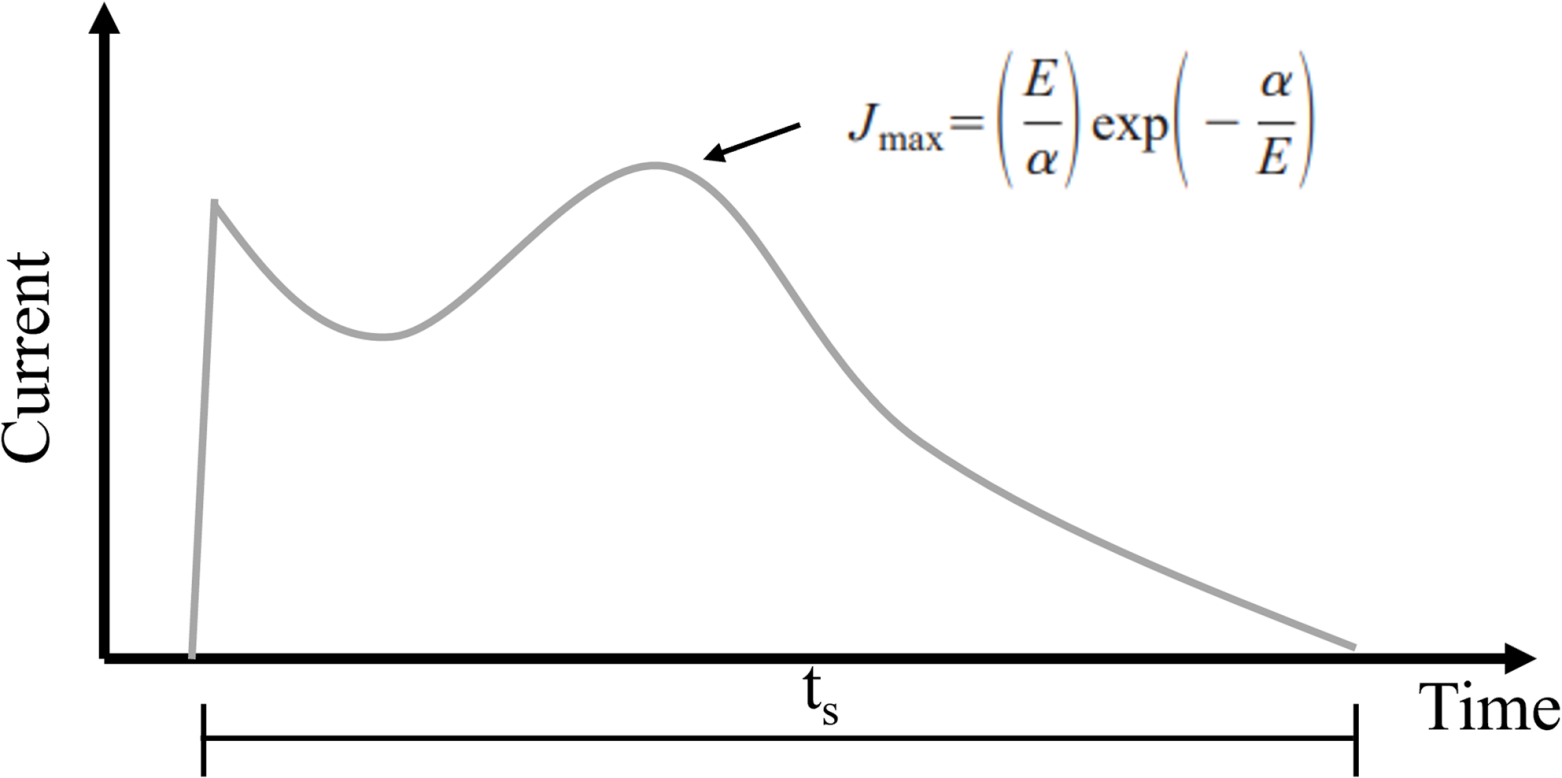
Schematic of the evolution of switching current with time during polarization reversal

In reversing the remanent polarization with a pulse directed in the opposite direction, there is a current evolution with time, as shown in Fig. 15. The shape of the switching current depends on the material, its orientation, temperature, and the applied electric field (E_app_). The schematic figure of the switching current as a function of time indicates a capacitive discharge at earlier times, followed by a current reversal associated with polarization. As indicated on the curve, there is a maximum current density and a total switching time, t_s_. These vary with the magnitude of the applied field, E_app_, and are related to the following relationships for the maximum switching current and the overall switching time [59, 76]:

5.1 $$ {\text{i}}_{\max } = {\text{ i}}_{\infty } \exp \left( { - \alpha /{\text{E}}_{{{\text{app}}}} } \right) $$

5.2 $$ {\text{t}}_{{\text{s}}} = {\text{t}}_{\infty } {\text{exp}}\left( { - \alpha /{\text{E}}_{{{\text{app}}}} } \right){\text{ or 1}}/{\text{t}}_{{\text{s}}} = {1}/{\text{t}}_{\infty } {\text{exp}}\left( {\alpha /{\text{E}}_{{{\text{app}}}} } \right) $$

where i_∞_ and t_∞_ are the current density and switching time for an infinite field strength, and α are constants that depend on temperature. At low fields, the switching time is primarily controlled by the nucleation rate, a process exponentially related to field strength [59]. At higher fields, the nucleation of new domains is no longer the rate-controlling process, which is then controlled by domain motion [59]. The linear part of the i_max_ is related to the movement of domains. It is noted that the reciprocal of the switching time is therefore related to the velocity, v, of the domain wall motion, which depends linearly on the applied field, E_app_ [76]:

5.3 $$ {1}/{\text{t}}_{{\text{s}}} = {\text{ v }} \approx \, \mu \left( {{\text{E}}_{{{\text{app}}}} - {\text{ E}}^{\prime } } \right) $$

where μ is the mobility of the domain wall and E′ is a limiting field similar to a coercive field strength. It separates the field associated with nucleation-controlled processes from those that are domain wall movement-controlled. To summarize, at high fields the switching current will be controlled by the rate limiting process of domain mobility that is linear, and at low fields the nucleation process controls the switching current magnitude and switching time.

**Fig. 16:**
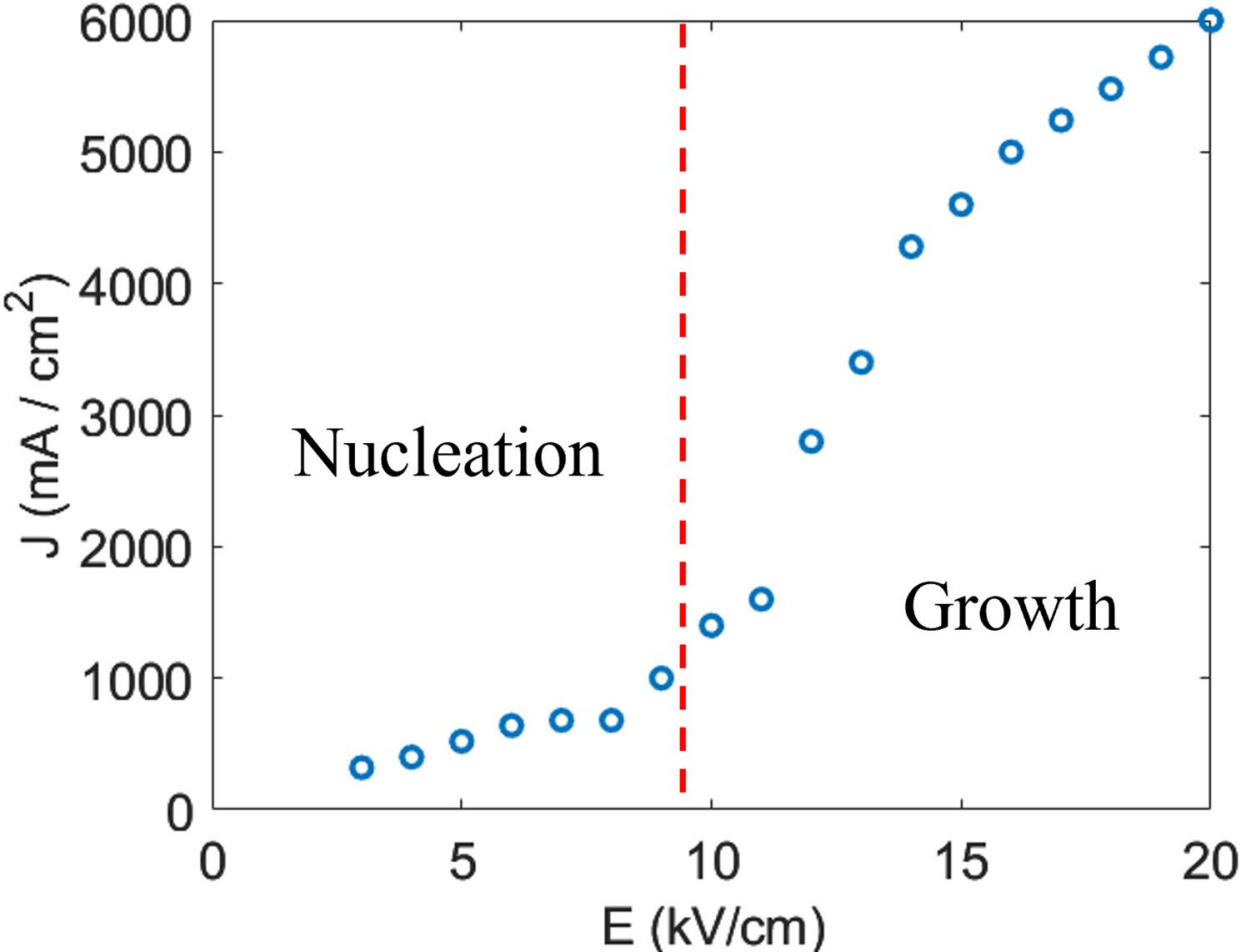
Current density vs. Electric field during switching in Mn: PIN-PMN-PT at room temperature, with nucleation limited and domain growth regions indicated

Shur et al. proposed a more sophisticated theoretical treatment of polarization switching based on a Kolmogorov-Avrami statistical theory [62]. However, the original Merz approach is sufficiently useful for determining the pulse electric field magnitudes to stimulate nuclei and later domain growth. Figure 16 shows the variation of the maximum current density, J_max_ (i_max_/area) versus applied electric field E_app_, and Table 4 shows the various switching parameters for different RFE crystals [61].

**Table 4:** α, μ, and E’ as functions of composition and orientation

Material:	α (kV/cm):	μ (cm^2^S^−1^ kV^−1^):	E’ (kV/cm):
PZN < 001 >	23.3	14.2	5.35
PZN < 111 >	15.7	4.89	4.25
PZN-4.5%PT < 001 >	8.9	23.7	5.10
PZN-4.5%PT < 111 >	6.71	9.98	4.28
PZN-8%PT < 001 >	2.74	9.57	4.09
PZN-8%PT < 111 >	2.27	5.11	3.97
Mn: PIN-PMN-PT < 001 >	9.9	5.71	6.5
Sm: PIN-PMN-PT < 001 >	7.76	4.48	5.1

The rate of domain wall mobilities is faster in the < 001 > directions relative to the < 111 > directions, and the compositions further away from the MPB (with a more relaxor-dominated character) have typically higher domain growth mobilities.

Beyond the high-field switching dynamics that control hysteresis and poling processes, domains are also important contributors to the dielectric properties in both poled and unpoled ferroelectric materials. The intrinsic/reversible and extrinsic/irreversible domain wall contributions under a small or intermediate AC field can be determined by monitoring the hysteretic response of the polarization and strain with the field in accordance with the Rayleigh equations [30, 77, 78]. These allow the piezoelectric coefficient, for example, to be expressed as [79]:

5.4 $$ {\text{d }} = {\text{ d}}_{{{\text{int}}}} + \alpha^{\prime \prime } {\text{E}}_{0} $$

where d is the piezoelectric coefficient, d_int_ is the initial piezoelectric coefficient that includes intrinsic and reversible domain contributions, α’’ is the Rayleigh Coefficient, and E_0_ is the field amplitude dependence of each sub-loop excitation. As an indicator of the extrinsic domain contribution to the piezoelectric properties, one can use the ratio α’’/d_int_. Similar expressions can be found for the polarization, which gives the relative permittivity in a Rayleigh equation as given by [21, 31, 79]:

5.5 $$ \varepsilon \, = \varepsilon_{{{\text{int}}}} + \alpha^{\prime } {\text{E}}_{0} $$

where ε_int_ is the intrinsic and reversible contributions to the permittivity, α’ is the permittivity Rayleigh coefficient, and E_o_ is the field amplitude.

**Fig. 17:**
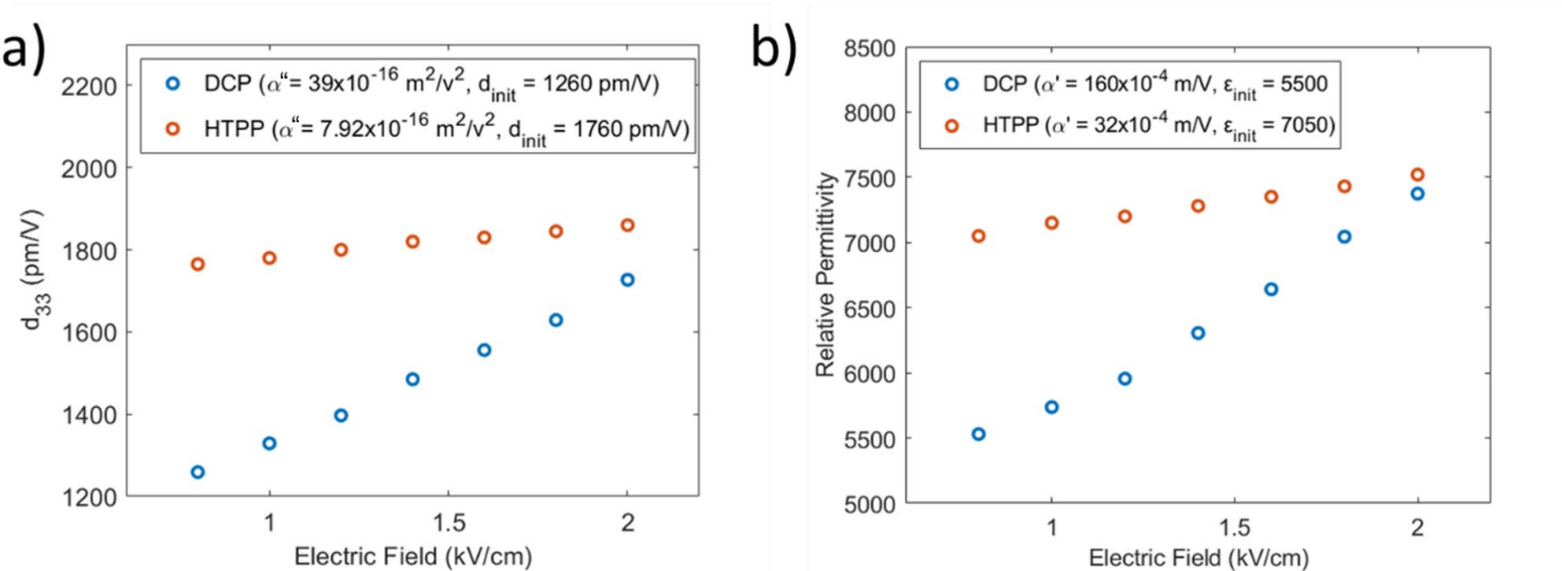
Rayleigh plots for (**a**) d_33_ vs. E for Sm: PIN-PMN-PT, and (**b**) ε_33_ vs. E for Sm: PIN-PMN-PT

Figure 17 shows a plot of the Rayleigh equations with their respective field dependence for DCP versus PP RFE [001] Gen II crystals. The slope represents the irreversible contribution to the property, and the intercept is the sum of the reversible and intrinsic contributions. The intrinsic portion is the structural contribution and is independent of the domains. In typical DC and AC poling, the major contribution is through the rotation of the polarization vector in the engineered domain state, but there are still extrinsic contributions that are believed to be important to the overall piezoelectric properties [29]. With PP materials, the Rayleigh ratio between the extrinsic/irreversible and intrinsic/reversible contributions is typically lower than that of DCP (an order of magnitude in some cases) [40, 65, 80, 81]. A comparison of current data and Rayleigh ratios found in the literature is given in Table 5 [82]. For both the single crystals in this study and previous investigations into polycrystalline PZT, soft materials often contain higher extrinsic contributions. In contrast, the Mn-doped systems suppressed the extrinsic/irreversible contributions to a high degree even in DCP. The impact that PP can have on these contributions can thus be best observed when comparing the Gen II crystals which do not initially depress domain mechanisms.

**Table 5:** Comparison of Rayleigh Ratios for PP and DC Poled [001] RFE and other Piezoelectric Materials

Material:	Microstructure:	Poling Procedure:	α’ / ε_init_ (× 10^–7^ m/V):
Mn: PIN-PMN-PT	Single Crystal	PP	2.00
Mn: PIN-PMN-PT	Single Crystal	DCP	3.00
Sm: PIN-PMN-PT	Single Crystal	PP	4.53
Sm: PIN-PMN-PT	Single Crystal	DCP	29.1
Mn: PMN-PZT	Textured Ceramic	PP	2.07
Mn: PMN-PZT	Textured Ceramic	DCP	3.70
Soft PZT	Random Poly	DCP	62.0
Hard PZT	Random Poly	DCP	25.0

We note that there is about an order of magnitude lower Rayleigh ratio in the PP relative to the DCP material in the < 001 > Gen II materials. Therefore, one would expect that the ability to phenomenologically model the intrinsic properties will be more accessible in the PP materials. If this is the case, it will provide researchers with a more effective way to model compositional and property trends and reduce extrinsic contributions from the properties in the textured ceramics. With the engineered domains that are locked into an RFE crystal < 001 > after DC and AC poling, the polarization variants can rotate, and this is a low hysteretic process. As could be seen from the Rayleigh analysis, there are still extrinsic irreversible contributions to the properties in the DC and AC cases, but these are reduced in the PP cases. This then provides the opportunity to model the properties of the PP with a Landau-Devonshire approach, as described below.

## The nature of RFE with PP in the FFE state

**Fig. 18:**
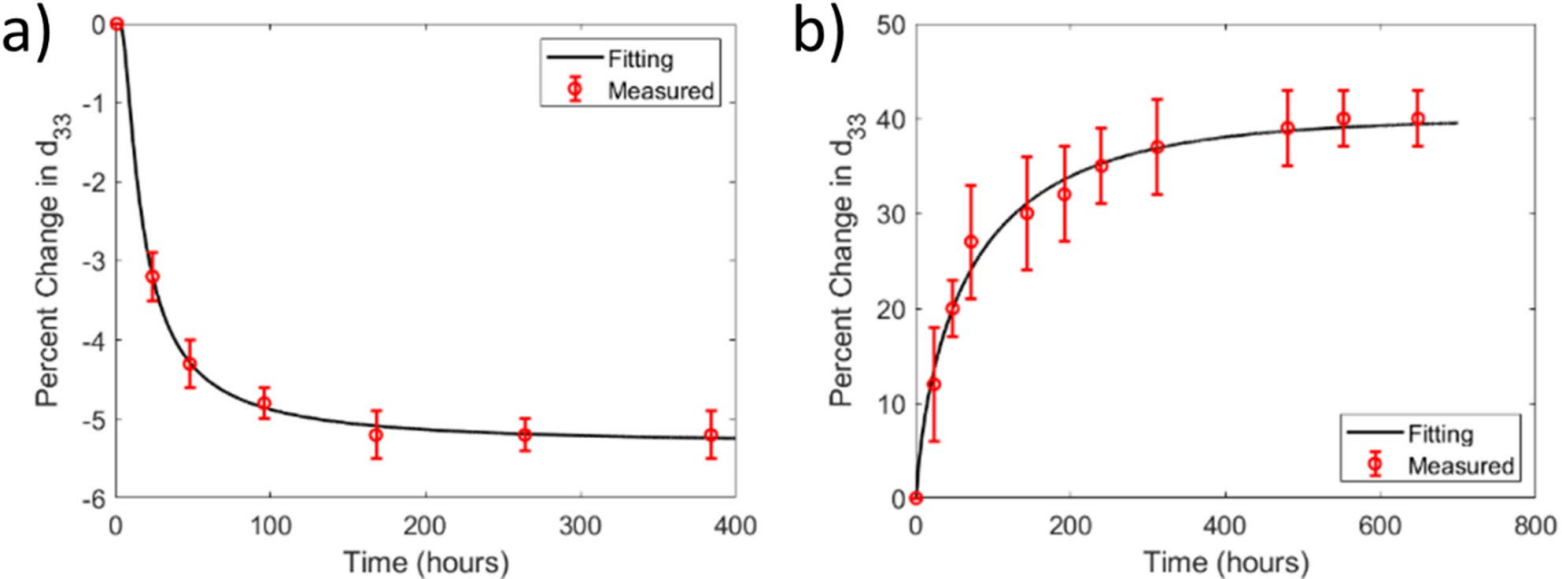
Room temperature aging of the piezoelectric coefficient as a function of time for a 1% Mn:PMN-PIN-PT crystal poled in [001] direction for (**a**) DCP and (**b**) PP

One of the most surprising consequences of the far-from-equilibrium state awarded by PP has been the positive aging of properties observed in hard RFE. When **Gen II** materials (soft materials like Sm-doped PIN-PMN-PT) are PP they show enhanced piezoelectric coefficients and permittivity [27, 45]. When PP a **Gen III** relaxor ferroelectric crystal or textured ceramic at elevated temperatures there is an unbalanced compensation of the depolarization field. This leads to the development of an internal electric field during the aging process, which in turn causes the electromechanical properties of the piezoelectric to increase with time [65]. This phenomenon, which is unique to this type of poling, is named positive aging [65]. Figure 18 shows a comparison of the d_33_ aging of a Gen III when poled with DCP and PP processes [65]. The property increase from the PP and aging process is associated with the intrinsic nature of the poled ferroelectric. Normal aging of properties for a ferroelectric follows:

6.1 $$d={d}_{\infty }+{d}_{1}exp\left[-{\left(t/\tau \right)}^{\beta }\right]$$

where *d*_*∞*_ is the time independent contribution, *d*_*1*_ is a constant, *t* is time, τ is the aging rate, and β is a constant between 0 and 1. The magnitude of β also determines whether there are short (β = 0.43) or long-range (β = 0.6) interactions during relaxation with a β of 1 being independent of any interactions at the so-called Debye limit [83, 84].

**Fig. 19:**
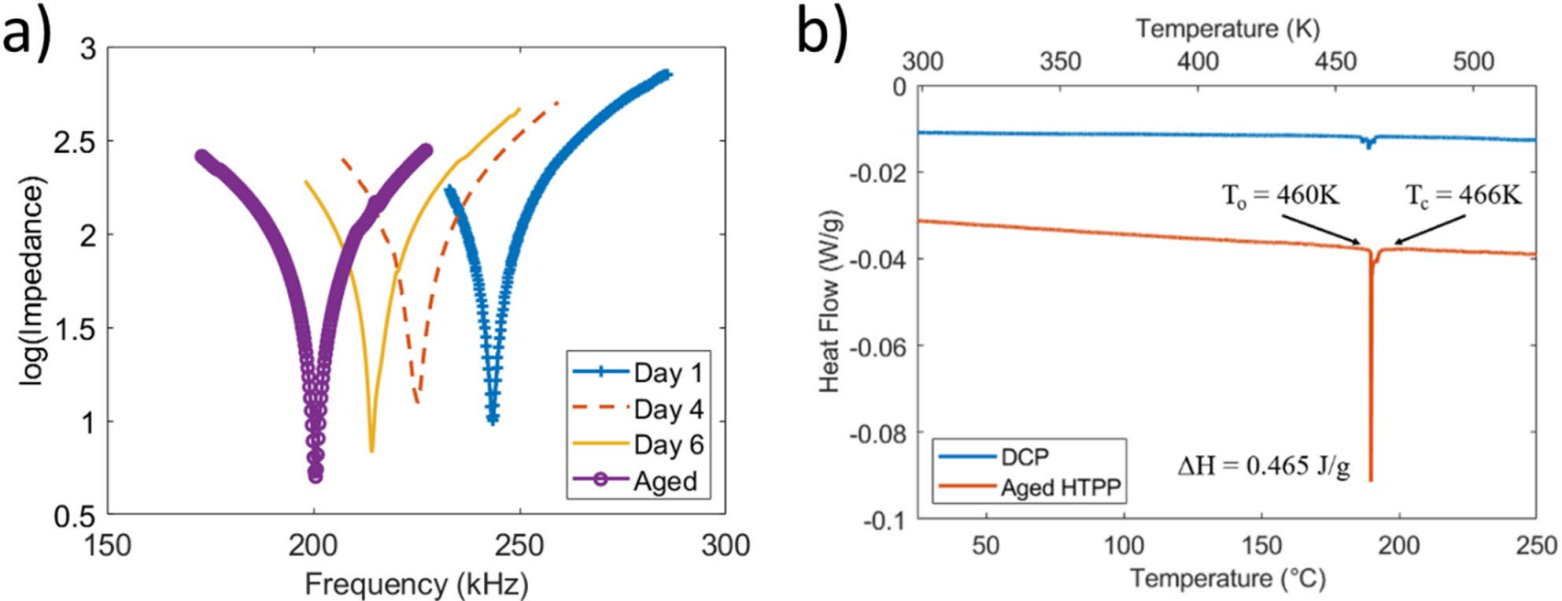
Changes in Mn: PIN-PMN-PT system following aging in HTPP for (**a**) resonance frequency and (**b**) latent heat

Aside from general property increases in the weeks following PP, these Gen III materials demonstrate further evidence of their FFE state. Figure 19 shows the changes to the elastic stiffness impacting the resonance frequency, and also a latent heat increase that arose in these hard samples poled via PP [65]. The decrease in resonance frequency with aging results in a more compliant matrix both compared to the day one case and DCP counterparts. Furthermore, the existence of a substantial latent heat peak at T_c_ is contrary to RFE behavior and suggests the creation of a first order phase transition. The increase in the latent heat is related to changes in the entropy that is kinetically trapped during PP and polarization alignment. The latent heat reflects the higher disorder that is likely associated with the dendritic domain structures, and this is released when it transitions to the more ordered state of the paraelectric phase.

There are very different properties and structural differences that arise with PP relative to conventional DCP and even ACP, and it is believed they are all associated with the fast kinetics of poling with pulse rise times on the order of microseconds. These property variations can be summarized in Table 6 below.

**Table 6:**
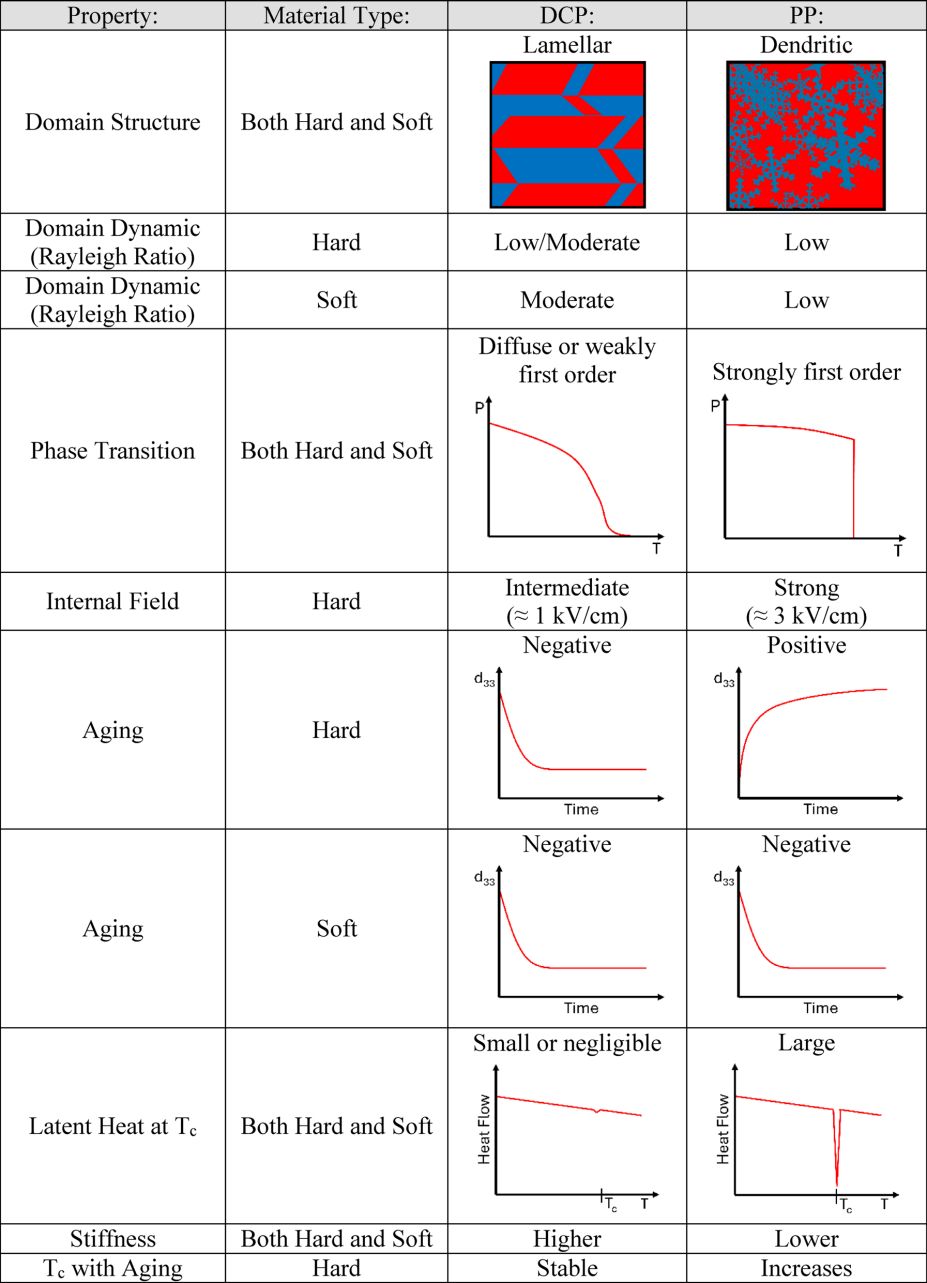
Property Trends with Hard and Soft Relaxor Materials via DCP and PP

## Landau-Devonshire thermodynamic potential and phenomenological modeling

**Fig. 20:**
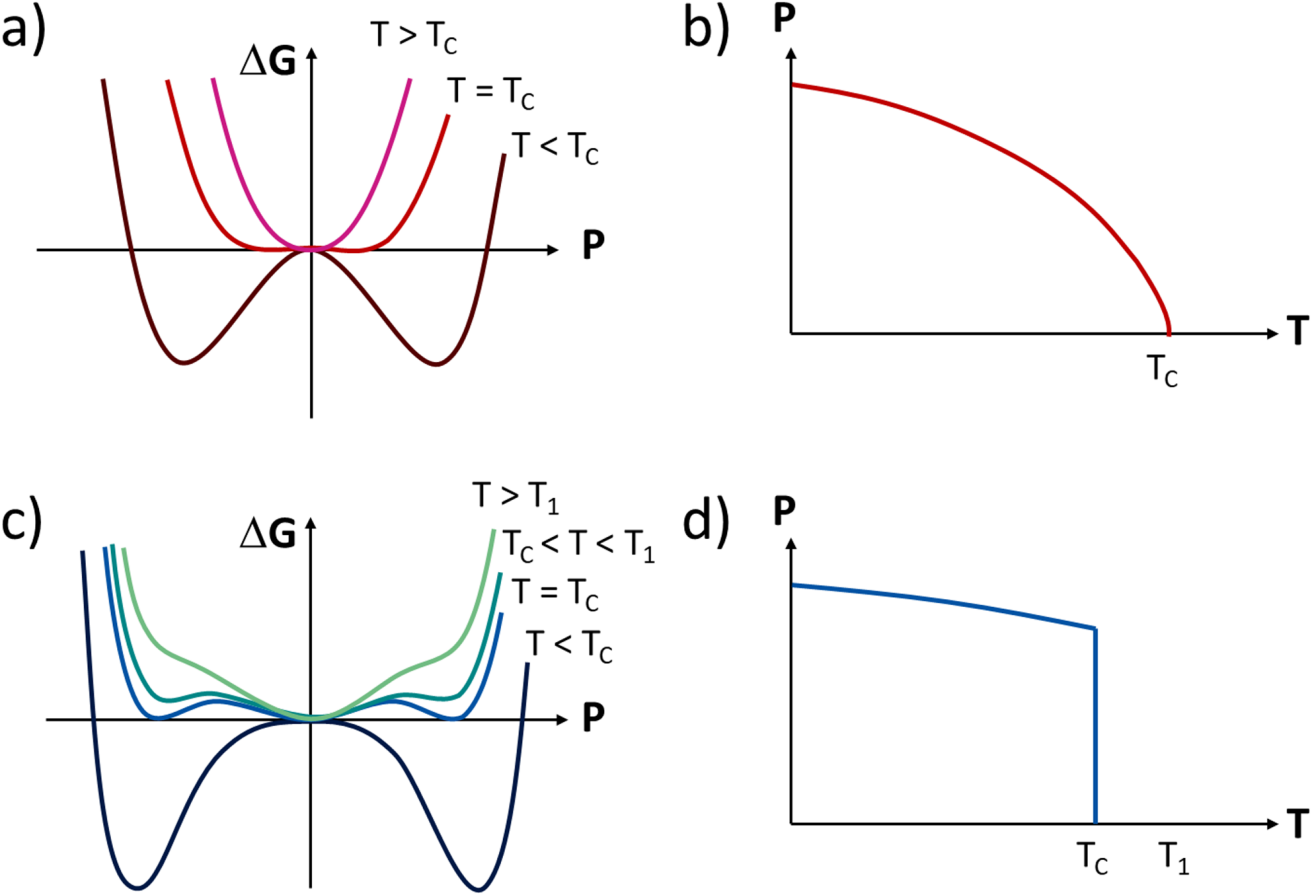
Comparison of Gibbs free energy (G) and polarization (P) as a function of temperature (T) across the paraelectric-ferroelectric transition: (**a**)–(**b**) for a second-order transition, (**c**)–(**d**) for a first-order transition

The Landau-Devonshire model is a well-known thermodynamic approach that considers ferroelectric material as a continuum and describes the free energy potential in the form of a polynomial with the order parameter of polarization [85]. This polynomial is symmetric with respect to the polarization directions. The quadratic coefficient is linearly temperature-dependent and related to the phase transition and the Curie temperature dependence of the permittivity. The quartic coefficient is related to the order of the phase transition (first order is negative, zero for tricritical, and second order is positive). The sextic term is often ignored in a second-order phase transition but is used in tricritical and first-order cases where it is positive. In ferroelectric materials, the elastic coupling to polarization is also of critical importance, meaning that the electrostrictive coupling of polarization and strain is important to fully describe the ferroelectric and its response to experimental variables such as temperature, electric field, and stress [86–88]. This is best addressed by expressing the Landau-Devonshire model in the form of the Gibbs Elastic Free Energy, G(T, X, P), as shown by [65, 89]:

7.1 $$ \begin{aligned} G\left( {T,X,P} \right) = & \frac{1}{2}\left( {\alpha + \frac{{2Q}}{{c^{E} }}X} \right)P^{2} \\ & + \frac{1}{4}\left( {\beta - \frac{{2Q^{2} }}{{c^{E} }}} \right)P^{4} \\ & + \frac{1}{6}\gamma P^{6} - \frac{{X^{2} }}{{2c^{E} }} \\ \end{aligned} $$

where G is the Gibbs elastic free energy, P is the polarization, X is the applied stress, c^E^ is the elastic stiffness under a constant electric field, and Q is the electrostrictive coefficient coupling strain and polarization. Figure 20 shows a comparison between the Gibbs Free Energy Function as a function of temperature through a PE to FE phase transition and the corresponding property variation for polarization as a function of temperature, for a second order or first order transition [90].

The coefficients in the thermodynamic function control the shape and position of the specific ferroelectric as it approaches the low-equilibrium energy state in the PE phase [90]. Typically, to account for the properties of an RFE, a more sophisticated phase field model is required, as was shown by Chen et al. [28, 91]. Under this model, the dynamics can be incorporated to describe domain configurations and properties. Typically, in RFEs, the dynamics and diffuse nature of the phase transition limit the use of the simple Landau-Devonshire theory [90]. It will be shown later that this is not the case under the PP FFE state of the RFE materials.

It should be noted that the coefficients can also be expressed with vectors and tensors to fully account for all the directional dependences of the crystals. The description of the thermodynamic free energy experience has been simplified in this case and will only be concerned with the macroscopic properties in the simple < 001 > directions.

Building from the properties of polarization and permittivity, the electromechanical properties and their intrinsic contributions can be determined by considering the electrostrictive coefficients. In general, electrostriction is present in all dielectric materials, regardless of their crystal symmetry. The strain (deformation), x, in electrostrictive materials is proportional to the square of the applied electric field or the electric polarization. While electrostriction is a universal phenomenon in dielectric materials, its effect is generally very small in most materials. However, relaxor ferroelectrics have higher electrostrictive constants, Q and M, and hence need to be considered with the piezoelectric properties of such materials. It should be noted that the properties are tensor properties; for the purpose of this demonstration, only the scalar connections between these properties will be considered to demonstrate the importance of the electrostrictive properties and the internal electric field, E_int_:

7.2 $$ {\text{x }} = {\text{ QP}}^{{2}} $$

where x is the strain, Q is the electrostrictive coefficient, and P is the polarization. Polarization is the sum of spontaneous polarization, P_s_, and the induced polarization, P_ind_. The induced polarization is given by P_ind_ = ε_o_ ε_r_ E, where ε_o_ is the permittivity of free space, ε_r_ is the relative permittivity, and E is the electric field. Here, one must consider the superposition of the applied electric field, E_app_, and the internal electric field, E_int_. It is important to observe that the M electrostrictive coefficient correlates the quadratic electric field with the strain as follows:

7.3 $$ {\text{x }} = {\text{ ME}}^{{2}} $$

With M = Q(ε_o_ ε_r_)^2^, this provides an important interconnection between the M and Q electrostrictive coefficients in high-permittivity materials such as ferroelectric relaxor materials.

Combining all these terms, an effective linear piezoelectric coefficient can be defined [65]:

7.4 $${d}_{\text{eff}}=\frac{dx}{dE}=2M{E}_{\text{int}}+2Q{\varepsilon }_{r}{\varepsilon }_{o}{P}_{s}$$

The existence of an internal field, E_int_, coupling with the M electrostrictive coefficient, provides an extra contribution beyond the intrinsic piezoelectric effect given by $2Q{\varepsilon }_{r}{\varepsilon }_{0}{P}_{s}$. Materials with larger internal fields are therefore an important consideration in the development of Gen III RFE materials and transducers.

Considering the piezoelectric coefficient measured with the direct Berlincourt and noting that there is thermodynamic equivalence between the converse and direct piezoelectric properties, these values are then compared to those derived using Eq. 7.4. This was done as a function of aging for the HTPP Mn doped crystals and textured ceramics, and also the Sm doped crystals, as summarized in Table 7 [65].

**Table 7:** Calculated and measured d_33_ for the Gen II and III PP [001] RFE crystals

Material:	Time:	d_33_ (pC/N), measured:	d_eff_ (pC/N), calculated:
Mn: PIN-PMN-PT	Day 1	990 ± 40	998 ± 65
Mn: PIN-PMN-PT	Aged	1,435 ± 55	1,422 ± 45
Mn: PMN-PZT	Day 1	651 ± 10	649 ± 35
Mn: PMN-PZT	Aged	740 ± 10	710 ± 20
Sm: PIN-PMN-PT	–	2,200 ± 100	2,340 ± 120

The increase in first order phase transition behavior, noted in Fig. 19, along with the changes seen in the elastic stiffness, c, and the increase in the electrostrictive coefficient, Q, are all consistent with the increase in the negative quadric coefficient in the Landau-Devonshire equation. The increase in the latent heat at the phase transition, T_c_ on heating is all predicted and consistent with the observed changes and thermodynamic predictions of the Landau-Devonshire model [92].

From the above Table 7**,** it is noted that the large increase in the piezoelectric property with the Gen III systems is strongly linked to the development of the internal field and the contribution of the *2ME*_*int*_ term in Eq. 7.4. With the Sm doped PIN-PMN-PT, there is no aging and no development of an internal field, and so only the second of the terms was required. Using Eq. 7.4, it is possible to predict the piezoelectric properties within 5% of the experimentally determined PP materials.

We can also extend the phenomenological model to the voltage piezoelectric coefficient for the PP systems. In general, the voltage piezoelectric coefficient, *g* that is defined by [75]:

7.5 $$ g = \, d/\varepsilon_{o} \varepsilon_{r} $$

Then, for the specific case of *g*_*33*_*,*

7.6 $$ g_{33 } = \, d_{33} /\varepsilon_{o} \,\varepsilon_{33} $$

With a generic intrinsic perspective, this gives the equation below that again has an enhancement due to a second term.

7.7 $$ g = 2QP_{s} + \, 2Q\varepsilon_{o} \varepsilon_{r} E_{{{\text{int}}}} $$

This additional contribution to the voltage piezoelectric coefficient develops under the aging relaxation process in conjunction with the formation of *E*_*int*_. It is possible that such changes account for the large g coefficients reported for the hard single crystals in Table 3. PP appears to suppress the dielectric response of the hard system while still being able to boost the piezoelectric coefficient. Similar to d_33_, it is possible to compare very well with the predicted phenomenological g_33_ value to the experimental value using Eq. 7.7. The results are given in Table 8.

**Table 8:** Calculated and measured d_33_ for the Gen II and III PP [001] RFE crystals

Material:	Time:	g_33_ (× 10^–3^ Vm/N), measured:	g_eff_ (× 10^–3^ Vm/N), calculated:
Mn: PIN-PMN-PT	Day 1	62.1 ± 5.7	64.5 ± 6.0
Mn: PIN-PMN-PT	Aged	58.1 ± 3.2	60.1 ± 5.3
Mn: PMN-PZT	Day 1	43.3 ± 1.5	46.5 ± 2.1
Mn: PMN-PZT	Aged	39.8 ± 1.1	43.2 ± 2.0
Sm: PIN-PMN-PT	–	25.9 ± 3.2	27.5 ± 3.5

## The future of pulse poling and the FFE state

This review highlights the early discoveries in pulsed poling (PP) as a promising processing method for ferroelectric single crystal and textured ceramic materials. Since the early studies of Yu and Randall, there have been few revisits to pulse poling. Most investigations of RFE materials have been on scaling up growth, developing new compositions, and exploring cost-effective strategies with TGG ceramics [43, 93]. Over the past decade, advancements such as ACP and water quench poling have sparked renewed interest in poling-based processes, and it is believed that PP offers new significant opportunities [45].

A deeper scientific understanding is needed to optimize PP, including the effects of rise rate, pulse holds, and the number of pulses. This requires evaluating different field amplitudes and temperatures, as well as considering material compositions and crystal orientations. Initial work focused on PP near the transition temperature between the rhombohedral (R) and tetragonal (T) phases. These methods should be expanded to a wider range of materials, including lead-free piezoelectrics, to better understand their behavior and domain structures. The study of dendritic domains, previously reported in LiNbO₃, should also be extended to all ferroelectric phases, including tetragonal materials [60]. Bulk screening, which is crucial for relaxation processes, necessitates a closer look at conductivity, doping, and non-stoichiometry in both donor- and acceptor-doped ferroelectrics. Such observations could be facilitated through in situ transmission electron microscopy (TEM) [94]. This technique would reveal how domain walls and preferred polarization directions propagate through a material during PP. Also, it could grant insight into how defects and other local inclusions influence the switching behavior when subjecting the sample to such swift fields.

The PP process must also be considered in relation to the specific form of the piezoelectric material, whether it’s textured ceramics, random ceramics, or thin films with either textured or epitaxial growth. It’s essential to understand the nucleation and growth processes, as well as the resulting domain structures, within textured ceramics that use different seeds and varying degrees of texture.

As the understanding of the poling (PP) process in ferroelectric materials evolves, particularly with respect to the domain structures and the ferroelectric symmetry distribution under different pulsing kinetics and temperature conditions, it will be possible to construct spatial and temporal models of the process. Techniques such as phase field modeling, which is based on the time-dependent Landau-Devonshire-Ginzburg (LDG) equation given below, will be essential for this [89]:

8.1 $$ \frac{\partial P}{{\partial t}} = - {\text{M}}\frac{\delta G}{{\delta P}} + \xi \left( {x,t} \right) $$

Here, M is the kinetic mobility, δG/δP is the variational derivative of the Gibbs free energy (7.1), and ξ is a stochastic noise term that accounts for thermal fluctuations and defects. The inclusion of this noise term introduces the random initial perturbations that lead to the complex, non-repeating, chaotic dendritic domain patterns.

From this modelling the formation of dendritic ferroelectric domains in the far from equilibrium state will spurn great interest. When a ferroelectric material undergoes rapid switching and the associated charge screening process, the spontaneous polarization does not have enough time to align into a single, or uniform domain, which is the lowest energy and most ordered state. This is a complex dendritic structure, with its many different polarization orientations and gradients that will lead to a higher configurational entropy compared to the highly ordered, aligned domain state. The system is then trapped in a metastable state with greater disorder. This self-trapping limits the domain wall contributions to properties, the so-called extrinsic contributions. Understanding all this interplay within a phase field model will aid the optimization of the PP process.

Furthermore, the time stability of the dendritic domain pattern with its vast and intricate network of domain walls is of great interest. This large total domain wall area means the system has a high excess surface energy. This high energy makes the dendritic state thermodynamically unstable, driving the system to reduce its total domain wall area over time through a process known as coarsening. The kinetics of dendritic domain coarsening in both hard and soft compositions is another very fundamental investigation that needs to be considered within the FFE state. Likewise, studying the PP of ferroelectric textured materials with different Lotgering factors will provide insights into the role of grain boundaries, crystallographic texture, and the anisotropy of switching kinetics. The Lotgering factor measures the degree of crystallographic texture, or preferred orientation, in a polycrystalline material [19]. Such insights will also see if there are opportunities for PP in random polycrystalline ferroelectric materials.

Translating PP to industry and the need to scale up the PP process for larger samples presents additional challenges. Larger samples have higher capacitance which can slow the switching kinetics due to the increased charging time. There’s also the risk of sample ringing, where multiple pulses can cause local heating and drive acoustic vibrations into the surrounding oil. Strategies such as damping and vacuum poling will be important for mitigating these effects. Therefore, careful consideration of pulse size, pulse shapes, and duty cycles is crucial when transitioning the poling process from the lab to production.

Although PP is still a relatively new method compared to established techniques like ACP, it is hoped that this review will inspire further investigation and innovation, paving the way for new advancements in piezoelectric ferroelectric materials.

## Data Availability

Data reported in this study can be provided upon request from the corresponding author, Michael Mervosh.
